# Novel Role for ESCRT-III Component CHMP4C in the Integrity of the Endocytic Network Utilized for Herpes Simplex Virus Envelopment

**DOI:** 10.1128/mBio.02183-20

**Published:** 2021-05-11

**Authors:** Tiffany Russell, Jerzy Samolej, Michael Hollinshead, Geoffrey L. Smith, Joanne Kite, Gillian Elliott

**Affiliations:** a Department of Microbial Sciences, University of Surrey, Guildford, United Kingdom; b Department of Pathology, University of Cambridge, Cambridge, United Kingdom; University of Alabama at Birmingham; St. Jude Children’s Research Hospital

**Keywords:** CHMP4C, endocytic pathway, herpes simplex virus, human herpesviruses, morphogenesis, secretory pathway, syntaxin 10, vesicular trafficking

## Abstract

Enveloped viruses exploit cellular trafficking pathways for their morphogenesis, providing potential scope for the development of new antiviral therapies. We have previously shown that herpes simplex virus 1 (HSV1) utilizes recycling endocytic membranes as the source of its envelope, in a process involving four Rab GTPases. To identify novel factors involved in HSV1 envelopment, we have screened a small interfering RNA (siRNA) library targeting over 80 human trafficking proteins, including coat proteins, adaptor proteins, fusion factors, fission factors, and Rab effectors. The depletion of 11 factors reduced virus yields by 20- to 100-fold, including three early secretory pathway proteins, four late secretory pathway proteins, and four endocytic pathway proteins, three of which are membrane fission factors. Five of the 11 targets were chosen for further analysis in virus infection, where it was found that the absence of only 1, the fission factor CHMP4C, but not the CHMP4A or CHMP4B paralogues, reduced virus production at the final stage of morphogenesis. Ultrastructural and confocal microscopy of CHMP4C-depleted, HSV1-infected cells showed an accumulation of endocytic membranes; extensive tubulation of recycling, transferrin receptor-positive endosomes indicative of aberrant fission; and a failure in virus envelopment. No effect on the late endocytic pathway was detected, while exogenous CHMP4C was shown to localize to recycling endosomes. Taken together, these data reveal a novel role for the CHMP4C fission factor in the integrity of the recycling endosomal network, which has been unveiled through the dependence of HSV1 on these membranes for the acquisition of their envelopes.

## INTRODUCTION

During the process of virus envelopment, intracellular trafficking pathways are subverted to supply lipid membranes for virus wrapping and release. These membranes contain virus-encoded glycoproteins that play a role in attachment and fusion during virus entry. While all virus glycoproteins start their life in the endoplasmic reticulum (ER), the cellular sites to which they are subsequently transported and where envelopment occurs vary between virus families (1). Herpes simplex virus 1 (HSV1) is a large enveloped virus that has an intricate morphogenesis pathway termed the envelopment-deenvelopment-reenvelopment pathway (2, 3), in which capsids form in the nucleus and bud through the inner nuclear membrane as primary virions (2, 4), using virus-encoded machinery termed the nuclear egress complex (5). The primary envelope is lost by fusion with the outer nuclear membrane, releasing naked capsids into the cytosol (2, 3). As for cellular membrane proteins, HSV1 envelope glycoproteins are cotranslationally inserted into the ER and transported through the secretory pathway to the Golgi apparatus and plasma membrane (6), with free capsids acquiring their final envelope from a wrapping site within the cytoplasm that contains these glycoproteins. Previously, we defined a model in which HSV1 acquires its envelope from glycoprotein-containing endocytic membranes that have been recently retrieved from the plasma membrane (7) rather than the *trans*-Golgi network (TGN) as often cited (8–10) and in agreement with previous studies from others (11). In this model, virus egress would then occur through the natural recycling of these membranes to the cell surface. This model has been supported by more recent studies showing that glycoproteins must be transported to the plasma membrane and endocytosed prior to envelopment taking place (12, 13).

Using targeted small interfering RNA (siRNA) screening, four members of the Rab family of GTPases were identified as being important for HSV1 envelopment, including Rab1 in the early secretory pathway and Rab5 and Rab11 in the endocytic pathway (7, 13). Moreover, in one of the first demonstrations of a biological role, Rab6A was shown to be critical for the transport of virus glycoproteins from the Golgi apparatus to the plasma membrane, thereby providing a source of envelope proteins for subsequent endocytic retrieval and wrapping (13). Rab GTPases function as central regulators of the four major steps of intracellular membrane traffic, vesicle budding, delivery, tethering, and fusion, to coordinate transport and delivery pathways (14, 15). However, they do not work alone, as these steps involve multiple cellular factors that organize, recruit, provide direction, and target specific cargoes throughout the cell. The aim of this study was to pinpoint steps along the cellular transport pathways that are exploited by HSV1 to target its envelope proteins to the correct assembly sites, where intervention would be debilitating to the virus and potentially tractable to antiviral intervention. As such, we have screened an siRNA library targeted at a rationally selected set of 82 factors, including coat proteins involved in membrane curvature and budding (16), adaptor proteins that select cargo for vesicles (17), membrane fusion and fission factors (18), and Rab effector proteins that provide specificity in vesicle targeting (19). Eleven factors were identified whose depletion altered virus production >20-fold, which are spread across the early and late secretory pathways and the endocytic pathway. While several of these factors blocked virus infection at early stages of the virus life cycle prior to morphogenesis, one of these factors, the endosomal sorting required for transport complex III (ESCRT-III) component CHMP4C, was shown to localize to and be required for the integrity of the recycling endocytic network needed for the envelopment of HSV1, which is suggestive of a role for this protein in the scission of these endocytic membranes. Hence, this study has unveiled a novel function of CHMP4C in the cell, reinforcing the power of using virus morphogenesis to identify novel activities in cellular trafficking.

## RESULTS

### Targeted siRNA screening identifies host trafficking factors involved in HSV1 replication.

**FIG 1:**
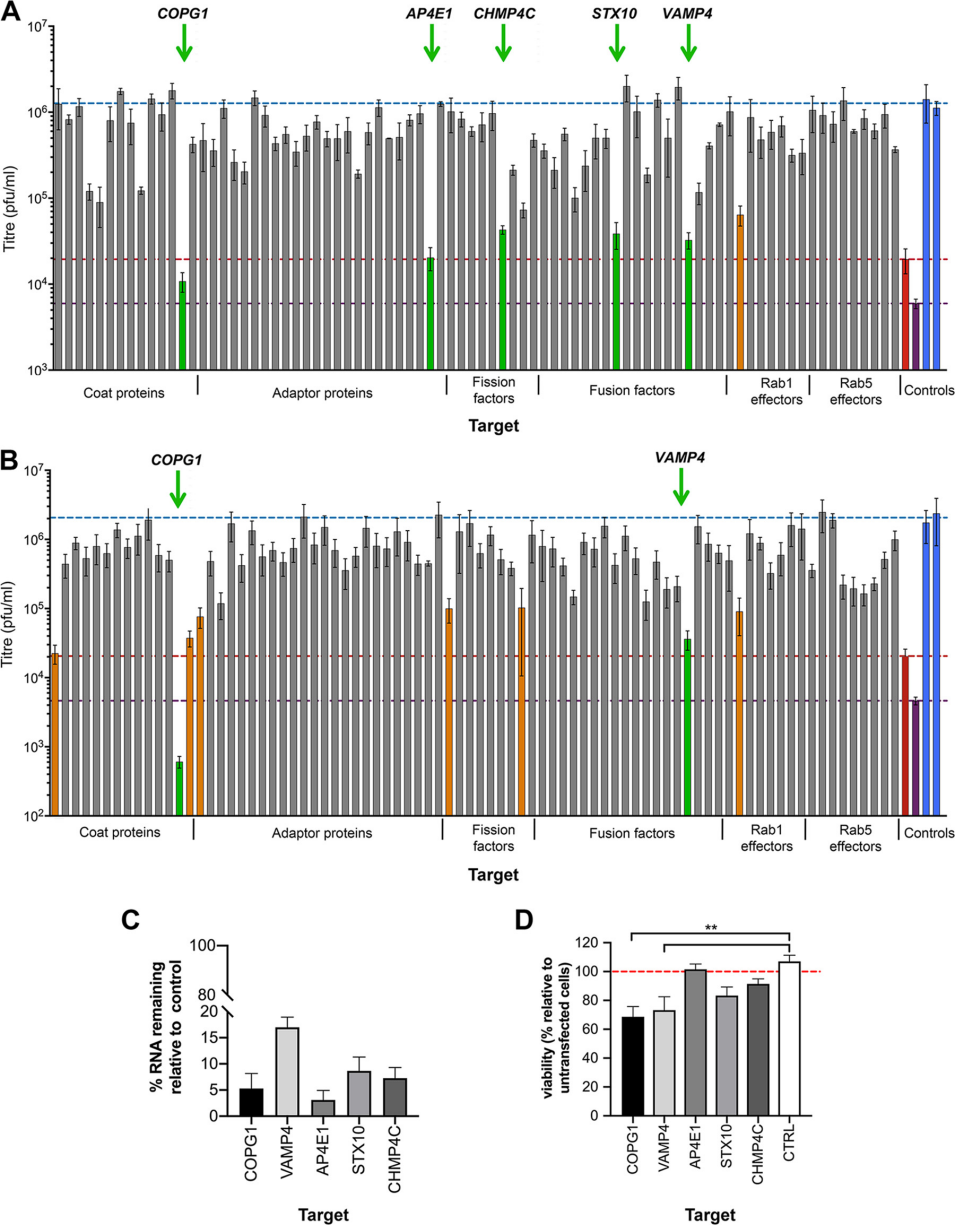
HSV1 replication in HeLa cells transfected with siRNA libraries against human trafficking proteins. (A and B) A total of 20 nM each siRNA from set 1 (A) and set 2 (B) was reverse transfected into HeLa cells in 96-well plates, incubated for 48 h, and then infected with HSV1 Sc16 at an MOI of 5 for 1 h. A gentle citrate buffer acid wash was performed to inactivate virus that had not penetrated the cells, before another 23 h of infection. Extracellular virus was harvested and titrated on Vero cells. The means ± standard errors of the means (SEM) are shown (*n *= 3). Blue, negative control; purple, nectin1; red, Rab6A; green, factors followed up in this study; orange, factors whose depletion caused a >20-fold reduction in titer but not followed up here. (C) siRNA transfection (set 1) was performed as a standard. After 48 h, cells were harvested for RNA isolation, and the percentage of mRNA remaining after knockdown was measured by RT-qPCR for the indicated targets (means ± SEM) (*n *= 3). (D) siRNA transfection was performed as a standard. After 48 h, cell viability was measured by a CellTiter-Glo assay, with viability expressed as a percentage relative to untransfected cells (means ± SEM) (*n *= 3). **, *P* < 0.01 (by one-way analysis of variance [ANOVA]).

Previously, siRNA library screening was used to identify three Rab GTPases involved in HSV1 envelopment (7, 13). To identify additional human cellular transport factors important for this process, we carried out a targeted siRNA screen directed against a range of host membrane trafficking factors, which included coat proteins, adaptor proteins, fusion factors, fission factors, and Rab effectors (see Table S1 in the supplemental material). Because the efficiency of library knockdown (KD) was anticipated to be between 70 and 85%, two individual sets of siRNAs against the 82 targets were used in independent screening experiments to reduce the chance of missing positive targets. The siRNAs were reverse transfected into HeLa cells for 48 h, before infection with HSV1. After 24 h of infection, the released extracellular virus was harvested from the medium and quantified by a plaque assay to compare the resulting yield of virus in siRNA-depleted cells to that in cells transfected with the negative siRNA control (CTRL) (Fig. 1A and B, blue). The depletion of the HSV1 entry receptor nectin1 (Fig. 1A and B, purple) (20) or the Rab GTPase Rab6A (Fig. 1A and B, red), which was shown to be critical for optimal HSV1 morphogenesis (13), served as a positive control. The average from three independent screens (Tables S2 and S3) revealed that the production of HSV1 was unaffected by the depletion of the majority of these host cell factors but that six siRNAs in set 1 and eight siRNAs in set 2 reduced virus yields by 20- to 100-fold (Fig. 1A and B, green and orange), with three of them being shared across the two sets. The identity of these 11 potential hits revealed three in the early secretory pathway (COPG1, COPG2, and GOLGA2), four in the late secretory pathway (AP1B1, AP4E2, VAMP4, and syntaxin 10 [STX10]), and four in the endocytic pathway (dynamin 2, clathrin light chain A, CHMP4C, and CHMP2A) (Table 1). Interestingly, three of the four endocytic proteins were fission factors involved in membrane abscission.

**TABLE 1:** Trafficking proteins implicated in HSV1 infection using siRNA libraries

Target	Relative yield	Fold drop	siRNA set(s)	Class	Function(s)
CLTA	0.01	100	2	Coat	Endocytosis, cytokinesis
COPG1	<0.01	>100	1, 2	Coat	Golgi-to-ER transport
COPG2	0.02	50	2	Coat	Golgi-to-ER transport
AP1B1	0.04	25	2	Adaptor	Transport from the TGN
AP4E1	0.03	33	1	Adaptor	Transport from the TGN
DNM2	0.05	20	2	Fission	Endocytosis, cytokinesis
CHMP4C	0.03	33	1	Fission	Endosomal sorting, cytokinesis
CHMP2A	0.05	20	1, 2	Fission	Endosomal sorting, cytokinesis
STX10	0.03	33	1	Fusion	Endosome-to-TGN transport
VAMP4	0.02	50	1, 2	Fusion	Endosome-to-TGN transport
GOLGA2	0.04	25	1, 2	Rab1 effector	Transport through the Golgi apparatus

10.1128/mBio.02183-20.4 TABLE S1 Genes for human trafficking factors targeted by the two siRNA libraries. Download Table S1, DOCX file, 0.02 MB. Copyright © 2021 Russell et al. 2021 Russell et al. https://creativecommons.org/licenses/by/4.0/ This content is distributed under the terms of the Creative Commons Attribution 4.0 International license.

10.1128/mBio.02183-20.5 TABLE S2 Relative virus yield from siRNA (set 1) library-transfected cells in three independent experiments. Download Table S2, XLSX file, 0.02 MB. Copyright © 2021 Russell et al. 2021 Russell et al. https://creativecommons.org/licenses/by/4.0/ This content is distributed under the terms of the Creative Commons Attribution 4.0 International license.

10.1128/mBio.02183-20.6 TABLE S3 Relative virus yield from siRNA (set 2) library-transfected cells in three independent experiments. Download Table S3, XLSX file, 0.01 MB. Copyright © 2021 Russell et al. 2021 Russell et al. https://creativecommons.org/licenses/by/4.0/ This content is distributed under the terms of the Creative Commons Attribution 4.0 International license.

Five of these 11 targets (Fig. 1A, green) were chosen for further analysis based on their greater effect on virus yield and their involvement in diverse stages of protein trafficking and membrane remodeling: COPG1, a subunit of the COP1 coatomer complex which coats vesicles moving retrograde between Golgi stacks and from the Golgi apparatus to the ER (21); AP4E1, a component of the AP4 adaptor complex found on some non-clathrin-coated vesicles leaving the TGN (22, 23); STX10 and VAMP4, SNARE proteins involved in vesicle fusion with target membranes (24, 25); and CHMP4C, a core component of the ESCRT-III machinery, whose only definitive function to date is at the final stages of cytokinesis (26). Reverse transcription-quantitative PCR (RT-qPCR) was used to confirm that for all five targets of interest, the knockdown from the first set of siRNAs was >80% at the transcript level (Fig. 1C). Given their potential importance for the cell, and to ensure that the requirement for each of these targets during virus infection was not a consequence of a pleiotropic and indirect effect of their depletion, a cell viability assay was also performed on depleted cells. This revealed that only the depletion of COPG1 or VAMP4 resulted in a significant reduction in cell viability (Fig. 1D), and of these two, only COPG1-depleted cells showed obvious signs of morphological changes (not shown), in line with results from a previous study (27).

**FIG 2:**
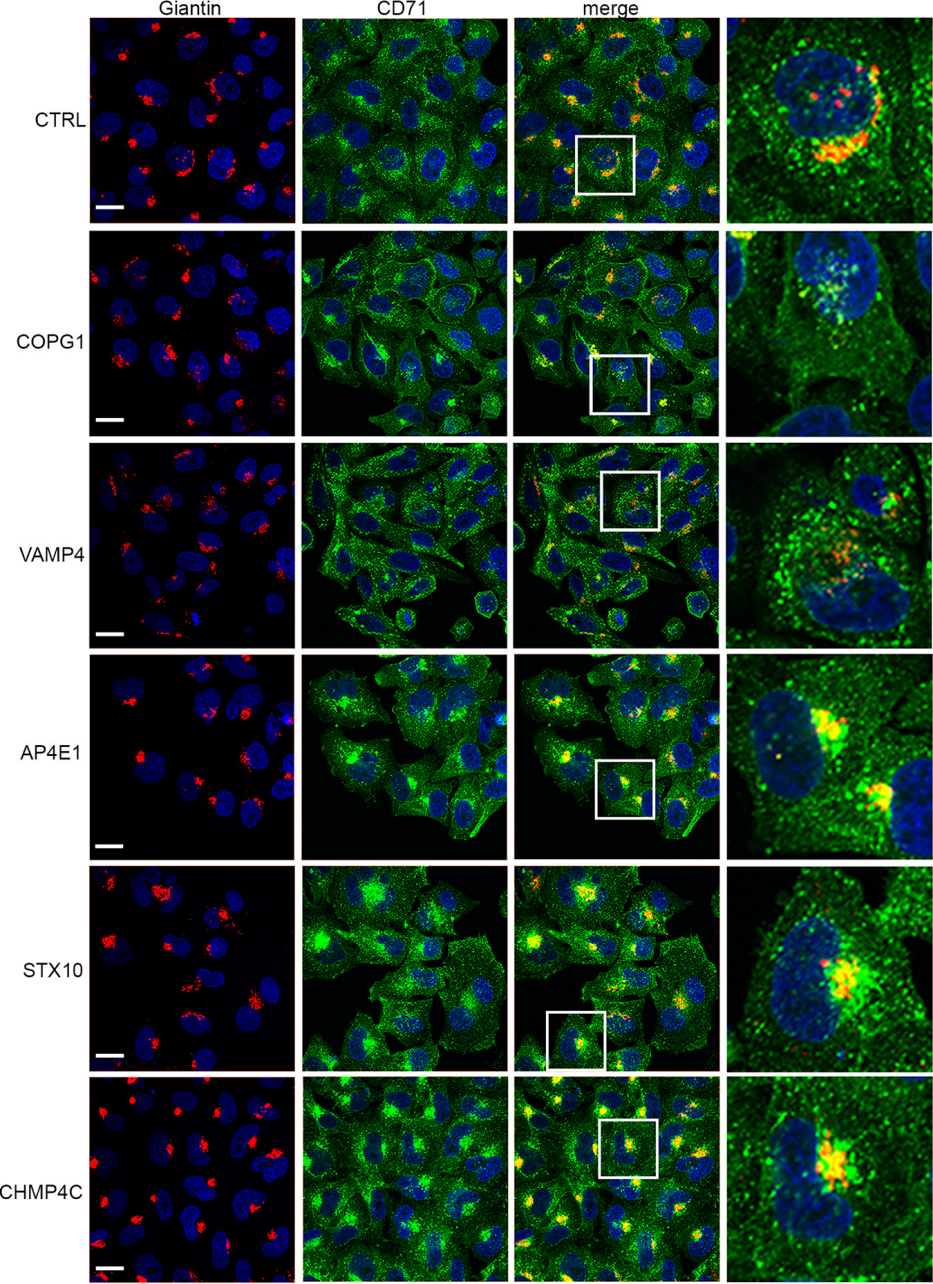
Integrity of secretory and endocytic pathways following siRNA depletion of target trafficking factors. HeLa cells transfected with negative siRNA (CTRL) or siRNAs for COPG1, VAMP4, AP4E1, STX10, and CHMP4C were fixed after 48 h, permeabilized, and stained with antibodies for the Golgi apparatus (giantin [in red]) and transferrin receptor (CD71 [in green]). Nuclei were stained with DAPI (blue). Bars = 20 μm.

To determine the effect of the depletion of each of these factors on the global appearance of the secretory and endocytic pathways, siRNA-transfected HeLa cells were costained for the Golgi marker giantin and the transferrin (Tf) receptor (CD71) marker for recycling endocytic membranes. Compared to control cells, the integrity of the Golgi apparatus was compromised in many of the COPG1-depleted cells, as might be anticipated from the role of COPG1 in retrograde vesicle transport through the Golgi apparatus (Fig. 2A, giantin staining). Likewise, the depletion of VAMP4 resulted in Golgi fragmentation, while the depletion of AP4E1, STX10, or CHMP4C had little effect on the steady-state appearance of the Golgi apparatus. Intracellular staining for transferrin receptor showed that as for the Golgi apparatus, the recycling endosomal network was compromised in COPG1-depleted cells (Fig. 2A, CD71 staining), and the depletion of VAMP4 resulted in altered transferrin receptor localization throughout the cytoplasm. In cells depleted of the other three target factors, there was a noticeable increase in the concentration of CD71 next to the nucleus, implying accumulation at the endocytic recycling compartment (ERC) which localizes around the microtubule-organizing center (MTOC) (28).

### Delineating the stages of virus infection affected by trafficking factor depletion.

**FIG 3:**
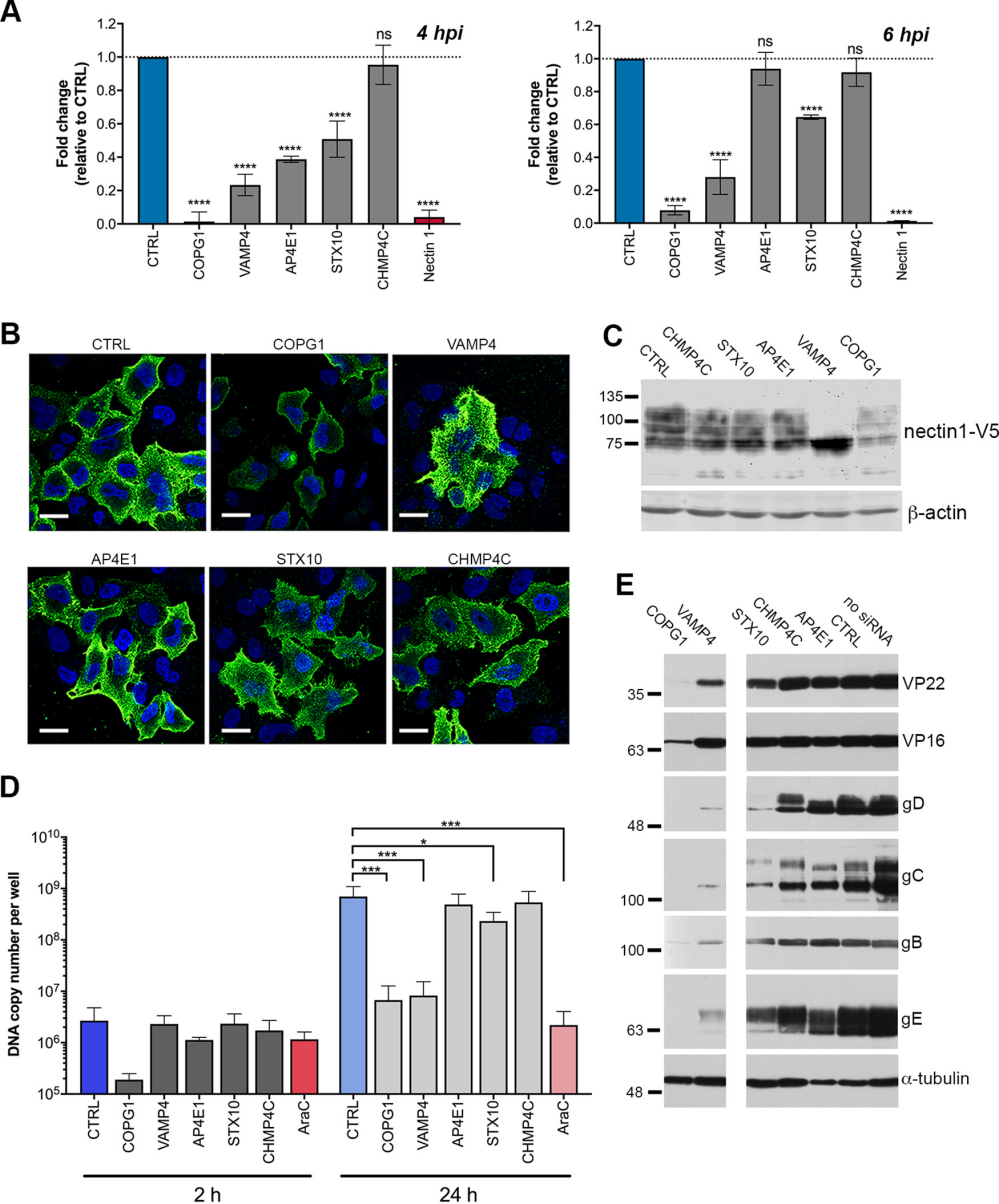
Depletion of target trafficking factors attenuates HSV1 replication at different stages of the virus life cycle. (A) HeLa cells transfected with siRNAs against five targets were infected 48 h later at an MOI of 5 with HSV1 IIE10*lacZ* for 4 h or 6 h, and β-gal activity was then measured by an *o*-nitrophenyl-β-d-galactopyranoside (ONPG) assay (means ± SEM) (*n *= 3). ****, *P* < 0.0001; ns, not significant (by one-way ANOVA). (B) HeLa cells were transfected with siRNAs against five targets together with a plasmid expressing V5-tagged nectin1. After 48 h, cells were fixed and stained with CK6 antibody without permeabilization to detect cell surface nectin1 (green) and nuclei stained with DAPI (blue). Bars = 20 μm. (C) Same as for panel B, but cells were harvested and analyzed by SDS-PAGE and Western blotting for nectin1-V5 and β-actin. (D) siRNA knockdown was performed, and cells were then infected with HSV1 Sc16 at an MOI of 2. DNA was isolated at 2 h or 24 h, and absolute qPCR was performed for gene *UL48* to determine the virus DNA copy number (means ± SEM) (*n *= 3). *, *P* < 0.05; **, *P* < 0.01; ***, *P* < 0.001 (by one-way ANOVA). (E) siRNA KD was performed, and cells were then infected with HSV1 Sc16 at an MOI of 5 for 16 h before harvesting and analysis by SDS-PAGE and Western blotting for the indicated proteins.

To establish where each of these identified factors was acting in the virus life cycle, we first determined if HSV1 entered cells with equivalent efficiencies after the depletion of each of the targets. To measure virus entry, reverse transfection of siRNAs was performed, and after 48 h, cells were infected synchronously with HSV1 expressing β-galactosidase (β-gal) under the control of the immediate early (IE) ICP0 promoter (Fig. 3A) (29). β-Galactosidase activity was measured as a surrogate for virus entry after 4 or 6 h (30), although strictly, this readout could be affected by any of the steps up to IE gene expression (virus binding, membrane fusion, delivery of capsids to the nucleus, and transcription of IE genes). Strikingly, the depletion of COPG1 significantly impaired expression from the ICP0 promoter, to a level similar to that with the depletion of the entry receptor nectin1, suggesting that COPG1-depleted cells may be defective for virus entry or one of the above-mentioned steps. Of the other four, only CHMP4C-depleted cells were able to support IE gene expression to the same level as that of control cells, with STX10-, AP4E1-, and VAMP4-depleted cells expressing between 2- and 5-fold less β-galactosidase than control cells after 4 h (Fig. 3A, 4 hpi [h postinfection]). Nonetheless, after 6 h, the β-galactosidase expression level in AP4E1-depleted cells had reached the level of the control cells, whereas STX10- and VAMP4-depleted cells still exhibited a lower level of β-galactosidase expression (Fig. 3A, 6 hpi), suggesting that the depletion of AP4E1 had slowed the early stages of virus infection, but this was eventually overcome.

Considering the potential for the involvement of these factors in the delivery of the nectin1 receptor to the cell surface and, therefore, an indirect involvement in virus entry, the effect of their depletion on the localization of nectin1 at the plasma membrane was tested. The available nectin1 antibodies were not sensitive enough to detect endogenous protein in HeLa cells, and hence, a plasmid expressing nectin1 tagged at its C terminus with the V5 epitope was first constructed and tested for its expression by transient transfection of HeLa cells (Fig. S1). Western blotting indicated that nectin1-V5 was expressed as a range of high-molecular-weight forms (Fig. S1A), which resolved to a lower-molecular-weight doublet following peptide-*N*-glycosidase F (PNGase F) treatment (Fig. S1B), indicating that nectin1 was multiply glycosylated, as expected from its eight predicted N-glycosylation sites (31). Moreover, immunofluorescence of nonpermeabilized, nectin1-V5-expressing cells with the anti-nectin1 antibody (Fig. S1C) confirmed the efficient cell surface localization of nectin1-V5. Staining of nonpermeabilized, nectin-V5-expressing cells that had been transfected with siRNAs to the five targets indicated efficient cell surface localization of nectin1 in all except COPG1-depleted cells (Fig. 3B). However, Western blotting of transfected cell extracts revealed that this low level of cell surface nectin1 in the absence of COPG1 correlated with overall reduced nectin1 expression, further indicating the limited integrity of cells depleted for COPG1 (Fig. 3C, COPG1). Interestingly, nectin1 expressed in the absence of VAMP4 was poorly glycosylated (Fig. 3C, VAMP4), suggesting that although the depletion of VAMP4 did not affect its transport to the plasma membrane, the structure of nectin1 encountered by the virus upon binding the cell would be altered, providing a possible explanation for the apparent reduction in virus entry in VAMP4-depleted cells.

10.1128/mBio.02183-20.1 FIG S1 Transient expression of V5-tagged constructs used in this study. (A) HeLa cells were transfected with plasmids expressing V5-CHMP4C, V5-STX10, or nectin1-V5 and analyzed 16 h later by SDS-PAGE and Western blotting with antibodies to the V5 epitope and α-tubulin. (B) Same as for panel A, but nectin1-V5-transfected cells were harvested and deglycosylated with PNGase F prior to analysis by SDS-PAGE and Western blotting. (C) HeLa cells grown on coverslips were transfected with the nectin1-V5-expressing plasmid. Sixteen hours later, cells were either cell surface stained with antibody to the extracellular domain of nectin1 prior to fixation or fixed and permeabilized, followed by staining with the same antibody (green). Nuclei were stained with DAPI (blue). Bar = 20 μm. Download FIG S1, DOCX file, 0.6 MB. Copyright © 2021 Russell et al. 2021 Russell et al. https://creativecommons.org/licenses/by/4.0/ This content is distributed under the terms of the Creative Commons Attribution 4.0 International license.

Next, the efficiency of viral DNA replication in cells transfected with siRNAs for each of the targets was compared to that of negative-control siRNA transfection after infection with HSV1 (Fig. 3D). Samples were harvested 2 or 24 h after infection, and the viral DNA copy number was determined by absolute qPCR for virus gene *UL48* to reflect input viral DNA (2 h) or viral DNA replication (24 h). Cells were also treated with an inhibitor of HSV1 viral DNA synthesis, cytosine arabinoside (AraC), to serve as a positive control for inhibition of DNA replication. The depletion of AP4E1 or CHMP4C had no significant effect on DNA replication compared to the negative control, with a 1,000-fold increase in DNA over the course of infection, indicating that despite some reduction in very early events of virus infection, the depletion of these factors had little effect on genome replication (Fig. 3D, 24 h). Similarly, the depletion of STX10 had only a slight effect on HSV1 DNA replication. In contrast, the depletion of VAMP4 almost abolished DNA replication, while in COPG1-depleted cells, input DNA was reduced 10-fold compared to control cells, indicating that the greatly reduced ability of the virus to initiate immediate early gene expression was due to reduced entry of virus genomes into the cell (Fig. 3D). Nonetheless, a modest 40-fold increase in viral DNA between 2 and 24 hpi suggests limited viral DNA replication in those cells that were able to support infection. These DNA replication results were reflected in Western blotting for late proteins in siRNA-depleted, infected cell lysates (Fig. 3E). In line with our entry and DNA replication studies, few of the virus proteins tested were detectable in COPG1-depleted cells, confirming that this infection had been blocked at very early stages of infection. VAMP4-depleted cells expressed detectable but relatively low levels of glycoproteins gB, gC, gD, and gE, likely reflecting the lack of DNA replication seen in these cells. In contrast, all proteins tested were expressed to the approximate levels of control cells in CHMP4C- and AP4E1-depleted cells, confirming that the virus replication cycle was blocked late in infection in the absence of these factors. In the case of STX10-depleted cells, there was also evidence for reduced glycoprotein expression despite a normal level of DNA replication and expression of the tegument proteins VP22 and VP16 (Fig. 3E), indicating a complex phenotype of virus infection in these cells.

### Depletion of CHMP4C or STX10 alters the trafficking of recycling endosomes.

**FIG 4:**
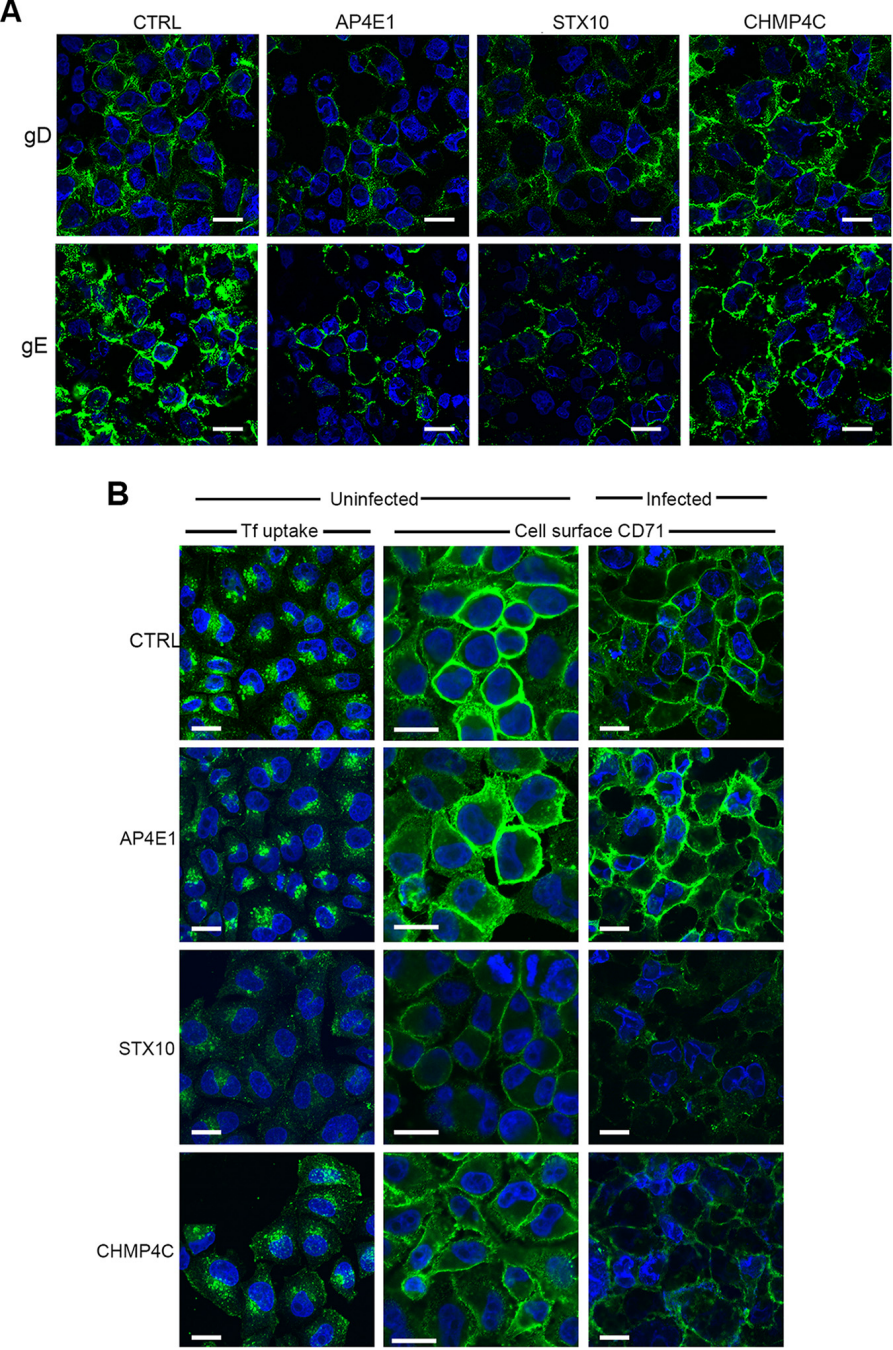
Cell surface localization of virus glycoproteins and transferrin receptor in siRNA-depleted cells. (A) siRNA knockdown was performed as indicated, and cells were then infected with HSV1 Sc16 at an MOI of 5 for 12 h before fixing and cell surface staining carried out for glycoproteins D and E (green) and staining of nuclei with DAPI (blue). Bars = 20 μm. (B) siRNA knockdown was performed as indicated. After 48 h, cells were incubated with Alexa Fluor 488-transferrin for 30 min and fixed (Tf uptake) or were fixed in the absence of transferrin, and cell surface staining was carried out for the transferrin receptor CD71 (uninfected and infected). Bars = 20 μm.

As the goal of this study was to identify factors involved in HSV1 morphogenesis, only AP4E1, STX10, and CHMP4C were taken forward from this stage. It was shown previously that HSV1 glycoproteins are transported to the cell surface prior to retrieval into wrapping membranes for virus envelopment (7, 13). Hence, to determine if glycoproteins can be delivered efficiently to the plasma membrane in AP4E1-, STX10-, and CHMP4C-depleted cells, the cell surface localization of glycoproteins gD and gE was examined by immunofluorescence of nonpermeabilized cells 12 h after infection in depleted and control siRNA-transfected cells. The cell surface levels of both glycoproteins were demonstrably lower in both AP4E1- and STX10-depleted cells than in the control (Fig. 4A), although it should be noted that for STX10, this is likely due to the overall reduction in glycoprotein synthesis as described above (Fig. 3E). In contrast, the levels in CHMP4C-depleted cells were comparable to those in control cells, suggesting that the depletion of CHMP4C inhibits virus production at a stage downstream of glycoprotein trafficking to the cell surface.

To determine the effect of the depletion of AP4E1, STX10, or CHMP4C on the overall appearance and activity of the endocytic pathway, uninfected cells depleted of each of these factors were tested for their ability to take up transferrin via the transferrin receptor. Fluorescent transferrin applied to cells is taken up into recycling endosomes via the transferrin receptor at the cell surface, whereupon it rapidly localizes to the ERC situated around the MTOC (Fig. S2A), demonstrating both the efficiency and kinetics of endosomal uptake. Importantly, this compartment is close to but does not overlap the TGN (Fig. S2B), indicating that these membranes are separate from this late secretory compartment. In line with its apparent role in TGN-to-plasma membrane transport, the depletion of AP4E1 had no effect on transferrin uptake compared to the control siRNA-transfected cells (Fig. 4B, Tf uptake), a result that correlated with the comparative cell surface CD71 staining of AP4E1-depleted and control transfected cells (Fig. 4B, uninfected, cell surface CD71). In contrast, the depletion of STX10 had a profound effect on Tf uptake compared to control cells (Fig. 4B, Tf uptake, STX10). This reduction in uptake correlated with a greatly reduced level of transferrin receptor at the cell surface (Fig. 4B, uninfected, CD71), in line with a previous study of STX10-depleted cells (32). In the case of CHMP4C depletion, transferrin uptake was altered compared to control cells, with transferrin-positive structures distributed throughout the cytoplasm and at the cell surface (Fig. 4B, Tf uptake, CHMP4C). This alteration reflected a partial reduction in transferrin receptor at the cell surface (Fig. 4B, uninfected, CD71, CHMP4C), indicative of an altered behavior of the endocytic recycling pathway in the absence of CHMP4C. The relative levels of cell surface transferrin receptor in uninfected cells were recapitulated in HSV1-infected cells, with the reduction in cell surface CD71 in the absence of CHMP4C being more pronounced in infected cells than in uninfected cells (Fig. 4B, cell surface CD71, infected). To determine if the depletion of CHMP4C had an effect on other stages of the endocytic pathway, cells that had been transfected with siRNAs for CHMP4C were also stained for the lysosomal marker lysosome-associated membrane protein 2 (LAMP2), the multivesicular/late endosomal marker CD63, or the TGN-to-endosome marker mannose-6-phosphate receptor (M6PR). In all cases, there was no change in the appearance of these compartments when CHMP4C was depleted (Fig. S3), indicating that CHMP4C depletion specifically alters the early and not the late endocytic pathway.

10.1128/mBio.02183-20.2 FIG S2 Uptake of transferrin into HeLa cells. (A) HeLa cells were incubated with Texas Red-conjugated transferrin for 30 min before fixing and staining for γ-tubulin (green) to label the MTOC. (B) HeLa cells were incubated with Alexa Fluor 488-transferrin (green) for 30 min before fixing and staining for TGN46 (red). Bar = 5 μm. Download FIG S2, DOCX file, 0.9 MB. Copyright © 2021 Russell et al. 2021 Russell et al. https://creativecommons.org/licenses/by/4.0/ This content is distributed under the terms of the Creative Commons Attribution 4.0 International license.

10.1128/mBio.02183-20.3 FIG S3 Depletion of CHMP4C has no effect on the late secretory pathway. HeLa cells were transfected with control or CHMP4C siRNAs and fixed and stained 2 days later for the lysosomal marker LAMP2, the late endosomal marker CD63, or the mannose-6-phosphate receptor (M6PR). Download FIG S3, DOCX file, 1.1 MB. Copyright © 2021 Russell et al. 2021 Russell et al. https://creativecommons.org/licenses/by/4.0/ This content is distributed under the terms of the Creative Commons Attribution 4.0 International license.

### CHMP4C but not CHMP4A or CHMP4B is required for HSV1 production.

**FIG 5:**
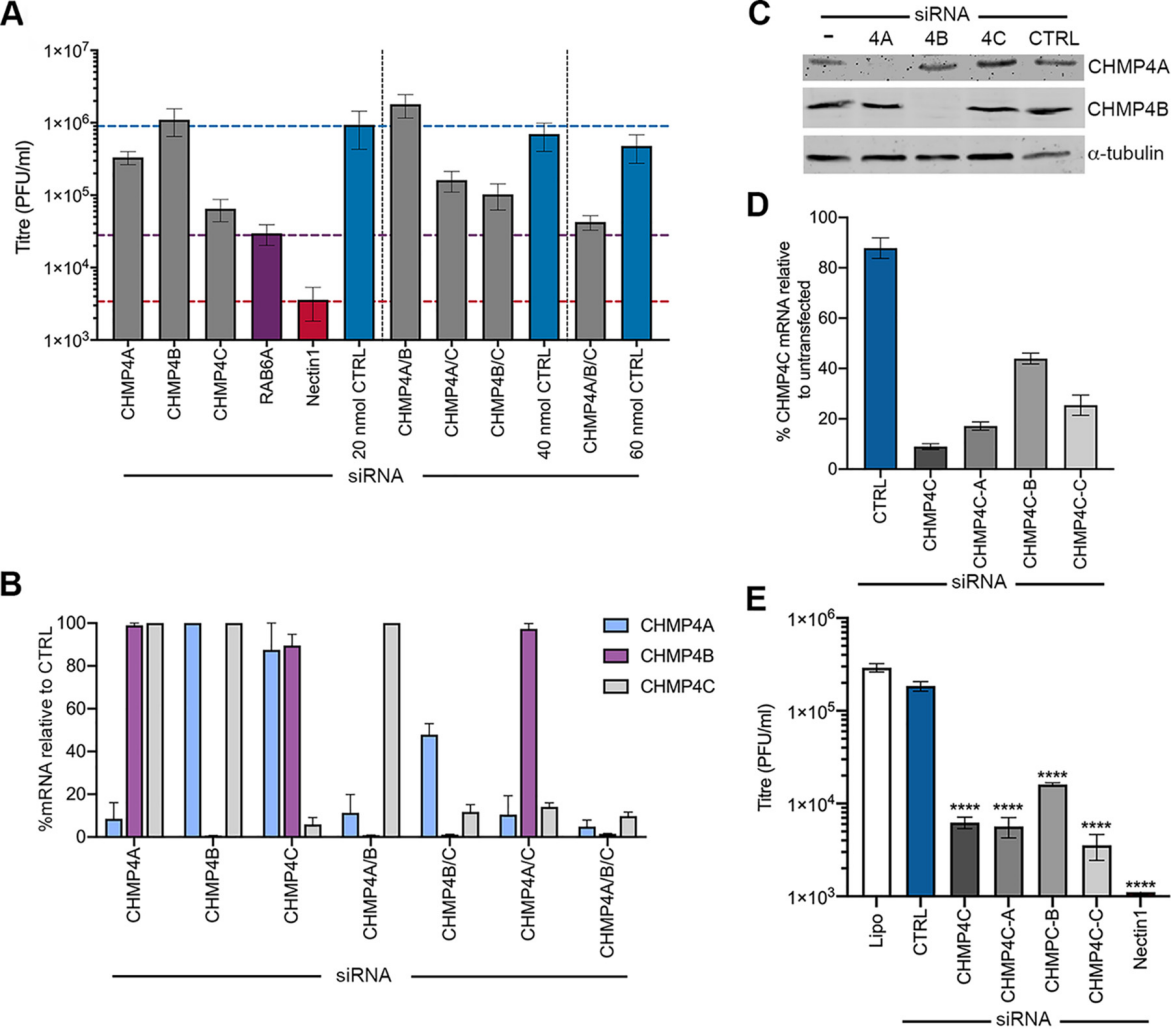
Depletion of CHMP4C but not CHMP4A or CHMP4B reduces HSV1 production. (A and B) A total of 20 nM each siRNA was reverse transfected alone or in combination as indicated, with controls transfected with equivalent amounts of control (CTRL) siRNA. (A) After 48 h, cells were infected with HSV1 Sc16 at an MOI of 5. Twenty-four hours later, extracellular virus was harvested and quantified by a plaque assay (means ± SEM) (*n* = 3). (B) After 48 h, cells were harvested for RNA isolation, and the percentage of mRNA remaining after knockdown was measured for CHMP4A, CHMP4B, or CHMP4C by RT-qPCR (means ± SEM) (*n *= 3). (C) HeLa cells were transfected with CTRL, CHMP4A, CHMP4B, CHMP4C, or no (−) siRNA; harvested 60 h later; and analyzed by SDS-PAGE and Western blotting for CHMP4A, CHMP4B, or α-tubulin. (D) HeLa cells were transfected with CTRL siRNA or four different CHMP4C siRNAs (CHMP4C, CHMPC-A, CHMP4C-B, or CHMP4C-C), harvested 72 h later, and analyzed by RT-qPCR for CHMP4C using 18S as a reference (means ± SEM) (*n *= 3). (E) HeLa cells were transfected as described above for panel D and then infected with HSV1, and 24 h later, extracellular virus was harvested and quantified by a plaque assay on Vero cells (means ± SEM) (*n* = 3). One-way ANOVA was carried out in relation to CTRL transfected cells. ****, *P* < 0.0001. Lipo, Lipofectamine without siRNA.

Collectively, these data indicate that CHMP4C depletion is alone among the five chosen factors in specifically blocking a very late stage of HSV1 infection, downstream of virus entry, genome replication, protein synthesis, and glycoprotein delivery to the plasma membrane. However, CHMP4C is one of three members of the CHMP4 family of proteins (33), and it has been suggested previously that all three members of this family may have a role in HSV1 morphogenesis (34). To confirm our results showing that CHMP4C and not CHMP4A or -4B is involved, as was implied from the initial screen, HeLa cells were transfected with siRNA for CHMP4A, CHMP4B, or CHMP4C, either alone or in combination, and infected with HSV1 after 48 h. Extracellular virus was harvested 24 h later and quantified by a plaque assay (Fig. 5A). The depletion of CHMP4A, CHMP4B, or CHMP4A and CHMP4B in combination did not reduce the amount of extracellular virus produced in comparison to cells transfected with the control siRNA. Furthermore, the reduction in the amount of virus produced by CHMP4C-depleted cells was not enhanced by depleting CHMP4A or CHMP4B in combination. RT-qPCR of RNA purified from HeLa cells that were depleted of CHMP4A, CHMP4B, or CHMP4C, either alone or in combination, was used to determine if transfection with multiple siRNAs affected the efficiency of siRNA knockdown (Fig. 5B). In all cases, transfection of the indicated siRNA resulted in a >90% decrease in transcript levels relative to the siRNA control. Furthermore, although we were unable to detect CHMP4C protein reliably by Western blotting, blotting of siRNA-transfected cell extracts confirmed that CHMP4A and CHMP4B proteins were both effectively and specifically depleted by their respective siRNAs (Fig. 5C). Finally, to confirm that CHMP4C depletion was not a consequence of an additional nonspecific off-target effect, we tested three more CHMP4C siRNAs for their ability to deplete the CHMP4C transcript (Fig. 5D) and inhibit HSV1 production in comparison to the original CHMP4C siRNA (Fig. 5E). All three new CHMP4C siRNAs (CHMP4C-A, CHMP4C-B, and CHMP4C-C) were shown to reduce the HSV1 yields by up to 50-fold, providing further evidence that the knockdown of CHMP4C affected the production of HSV1 (Fig. 5E). Altogether, these results suggest that the depletion of only CHMP4C, and not CHMP4A or CHMP4B, had a detrimental effect on HSV1 infection in our experimental system.

### CHMP4C localizes to recycling endosomes.

**FIG 6:**
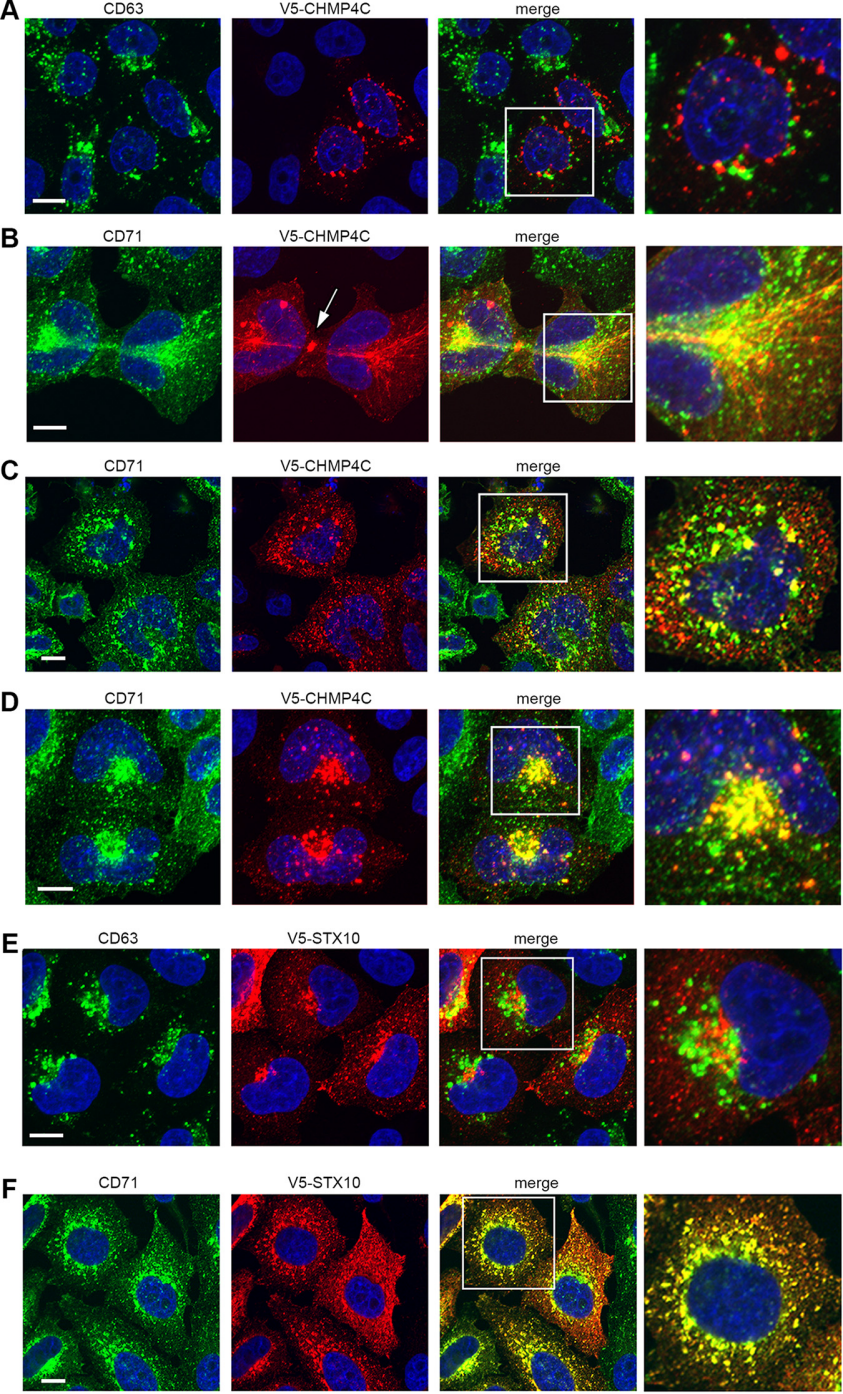
Exogenously expressed CHMP4C and STX10 colocalize with the transferrin receptor CD71. HeLa cells were transfected with a plasmid expressing V5-CHMP4C (A to D) or V5-STX10 (E and F) and after 24 h were fixed and stained for V5 (red) and CD63 (green in panels A and E) or CD71 (green in panels B to D and F). Nuclei were stained with DAPI (blue). Bars = 10 μm.

CHMP4C is a component of the ESCRT-III fission machinery that would be expected to be involved in membrane scission at various locations in the cell. It has been well characterized for its role in the abscission checkpoint during cytokinesis, where it localizes to the midbody ring to delay cytokinesis and protect against DNA damage accumulation (26, 35). Although less detail is available on its localization in the interphase cell, by extrapolation with other ESCRT-III proteins and their role in multivesicular body (MVB) biogenesis, where they play a role in the involution and scission of membranes budding into the lumen of these structures (reviewed in reference 36), it would be expected that at least a proportion would localize to the late endocytic pathway. Given the unexpected effect of CHMP4C depletion on recycling endosomes as noted above, its cellular localization in relation to recycling and late endocytic compartments was assessed next. Endogenous CHMP4C was not detectable with antibodies available to us, so we therefore examined cells expressing V5-tagged CHMP4C (with expression confirmed by Western blotting [Fig. S1A]), focusing on cells expressing lower levels of the protein. Unexpectedly, no colocalization with the MVB marker CD63 (Fig. 6A) was found, but a substantial overlap was observed with the transferrin receptor marker for recycling endosomes in cells at different stages of the cell cycle (CD71) (Fig. 6B to D). First, as described previously by many others, overexpressed CHMP4C localized to the midbody of cells in the process of cytokinesis (Fig. 6B, arrow), confirming that it localizes as expected even when expressed exogenously (26). In the same cells, a population of CHMP4C localized around the centrosomes and in tubules emanating from the centrosome. Likewise, recycling endosomes were also localized around the centrosomes together with the intercellular bridge, where they are known to deliver membrane to the cleavage furrow (37). In interphase cells, there was also a high degree of overlap between the CHMP4C signal and the transferrin receptor signal (Fig. 6C), which was particularly obvious in cells that were judged to be entering interphase shortly after cytokinesis (Fig. 6D).

Given the effect of STX10 depletion on CD71 localization to the plasma membrane (Fig. 2B) and its effect on virus replication, we also tested the localization of exogenously expressed V5-STX10 (confirmed by Western blotting [Fig. S1A]). As for CHMP4C, there was no overlap between V5-STX10 and CD63 (Fig. 6E), but there was almost total colocalization with the transferrin receptor (Fig. 6F), suggesting that this SNARE protein localizes to recycling endosomes. Although it has previously been reported that STX10 localizes to the TGN (24), our results showing colocalization with the transferrin receptor help to correlate the localization of this SNARE protein with its previously identified role, both here and elsewhere, in the recycling of the transferrin receptor to the plasma membrane (32).

### Depletion of CHMP4C leads to extensive accumulation and tubulation of endocytic membranes in HSV1-infected cells.

**FIG 7:**
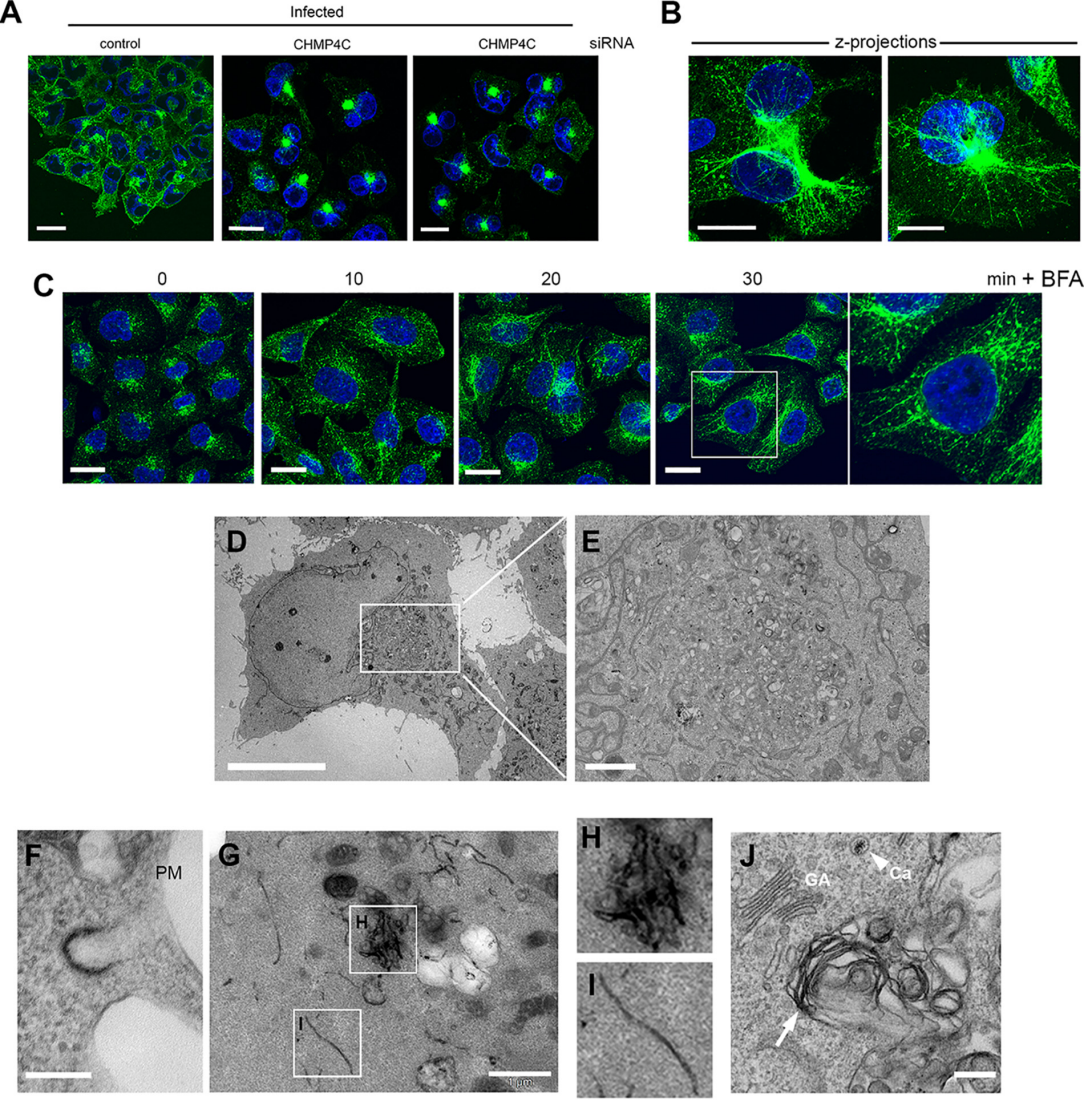
Depletion of CHMP4C leads to tubulation and accumulation of recycling endocytic membranes in HSV1 infection. (A) HeLa cells transfected with CTRL or CHMP4C siRNAs were infected with Sc16 for 12 h, fixed, and stained for transferrin receptor (CD71). Bars = 20 μm. (B) Same as for panel A, but individual CHMP4C-depleted cells are presented as z-projections of the entire cell volume. Bars = 20 μm. (C) HeLa cells were treated with BFA (1 μg/ml), fixed at the indicated times thereafter, and stained for the transferrin receptor (CD71). Bars = 20 μm. (D and E) TEM images of CHMP4C-depleted, HSV1 infected cells showing membrane accumulation in the center of cells. Bars = 10 μm (D) and 1 μm (E). (F to J) Same as for panel D, but cells were incubated with HRP-transferrin for 30 min prior to fixation and processing for TEM. (F) Section (120 nm deep) showing a clathrin-coated pit labeled with HRP-transferrin. Bar = 200 nm. (G to I) Sections (300 nm deep) showing HRP-transferrin-positive tubules in the cytoplasm. (J) Ultrathin (60-nm) section showing HRP-positive endocytic membranes (arrow) clustered close to the Golgi apparatus (GA). The arrowhead points to naked capsid (Ca) in the cytosol.

As described above, CHMP4C localizes with recycling endosomes, and its depletion demonstrably alters the behavior of the recycling endocytic pathway. Further analysis of the transferrin receptor in infected cells revealed that in the absence of CHMP4C, the transferrin receptor exhibited an altered localization comprising accumulation at the MTOC concomitant with a reduction at the plasma membrane (Fig. 7A), in line with the relative cell surface staining observed in infected cells as described above (Fig. 4). Upon closer examination, ∼25% of cells were found to exhibit long, thin, CD71-positive tubular extensions emanating from the region of the MTOC toward the cell periphery (Fig. 7B, z-projections). These tubules were reminiscent of data from previous studies on brefeldin A (BFA)-treated cells, where it has been shown that BFA, which inhibits Arf GTPases, inhibits coat assembly and the subsequent fission of endocytic membranes (38). Therefore, the staining pattern of CD71 seen in the infected CHMP4C-depleted cells was compared to that in BFA-treated cells, fixed at various times after addition (Fig. 7C). Under these conditions, the transferrin receptor was found in similarly thin, tubular extensions emanating from the MTOC, as described in previous studies (39).

Ultrastructural analysis by transmission electron microscopy (TEM) revealed massive membrane accumulations in the cytoplasm next to the nucleus of many infected cells depleted of CHMP4C (Fig. 7D and E). To further correlate the results with confocal microscopy of recycling endosomes, TEM of CHMP4C-depleted, HSV1-infected cells that had been incubated with horseradish peroxidase (HRP)-transferrin to specifically label transferrin receptor-positive recycling membranes was carried out. HRP-transferrin-positive clathrin-coated pits were identified (Fig. 7F), indicating the binding of transferrin to its receptor. Sectioning to a depth of 300 nm revealed the presence of extensive, HRP-transferrin-positive tubules either clustered (Fig. 7G and H) or individually running through the cytoplasm (Fig. 7G and I), confirming that these tubules formed by the depletion of CHMP4C were indeed a component of the transferrin receptor-positive recycling endocytic network. Moreover, large accumulations of HRP-positive membranes were detected in locations similar to those shown in Fig. 7D and E, suggesting that these endocytosed membranes ultimately ended up in aberrant membrane clusters within the central region of the cell (Fig. 7J). This perturbation of the recycling endocytic network was more pronounced in infected cells than that seen in uninfected cells in the absence of CHMP4C.

### Depletion of CHMP4C inhibits envelopment of HSV1.

**FIG 8:**
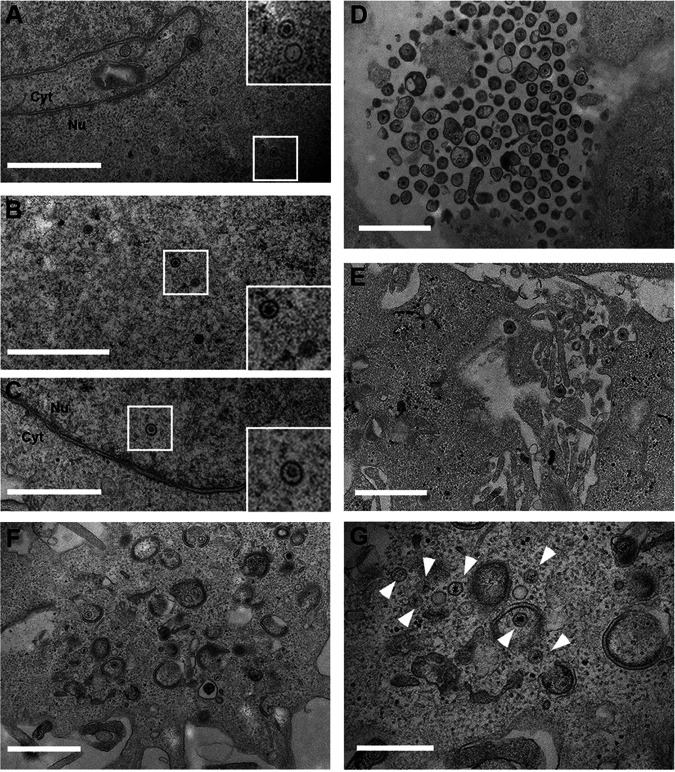
HSV1 envelopment is defective in CHMP4C-depleted HeLa cells. HeLa cells transfected with CTRL (A and D) or CHMP4C (B, C, and E to G) siRNAs were infected after 48 h with Sc16 at an MOI of 2 and 12 h later were fixed and processed for imaging by TEM. (A) Capsids in the nucleus (Nu) of CTRL siRNA-transfected infected cells. (B and C) Capsids in the nucleus of CHMP4C siRNA-transfected infected cells. (D) Abundant enveloped virions released from the cell surface of CTRL siRNA-transfected infected cells. (E) Enveloped virions at the cell surface of CHMP4C siRNA-transfected infected cells were rarely found. (F and G) Numerous cytoplasmic capsids were found either naked or associated with tubules, but fully enwrapped capsids were difficult to locate. Bars = 1 μm (A to F) and 500 nm (G). Arrowheads indicate capsids in the cytoplasm (Cyt).

To investigate how this effect of CHMP4C depletion on the endocytic network affects virus morphogenesis, we used TEM to examine CHMP4C-depleted, HSV1-infected HeLa cells at 16 h compared to control siRNA-transfected cells. In control and CHMP4C-depleted infected cells, newly assembled capsids were found inside the nucleus, confirming that capsid assembly occurs as normal in the absence of CHMP4C (compare Fig. 8A with Fig. 8B and C). In contrast, while many enveloped virions were found released from the control transfected cells (Fig. 8D), in CHMP4C-depleted infected cells, only a small number of enveloped virions were found outside the cell (Fig. 8E), in accordance with the results of our released virus quantification (Fig. 1A and Fig. 5A). Unlike the fully enveloped virions ordinarily found wrapped in double membranes in the cytoplasm of HSV1-infected cells (7), in CHMP4C-depleted infected cells, these fully wrapped capsids were much rarer and difficult to detect despite the presence of numerous capsids within the cytoplasm (Fig. 8F and G, arrowheads). Moreover, many capsids were found associated with incomplete double membranes, suggesting a failure to seal the membrane around the capsid (Fig. 8G, arrowheads). Taken together, these results indicate that the depletion of the fission factor CHMP4C alters the integrity of the recycling endocytic network, leading to a global accumulation of membranes at the ERC and abrogation of the final wrapping events in HSV1 morphogenesis. In short, this study identifies a new role for CHMP4C in the biogenesis of recycling endosomes.

## DISCUSSION

The basic replication strategies of viruses, and their exploitation of the host cells that they infect, must be understood before novel therapeutics can be developed. Many highly pathogenic human viruses are surrounded by lipid envelopes that are rich in virus-encoded proteins and are derived from intracellular membranes. These pathogens are therefore highly dependent on cellular trafficking pathways for the production of infectious virus. Indeed, many potentially share common routes of envelopment, raising the possibility of targeting envelopment as a broad-spectrum intervention strategy. This study focused on the alphaherpesvirus HSV1, a large enveloped DNA virus which has a complex morphogenesis pathway involving the nucleus, the cytoplasm, and many aspects of cellular membrane trafficking pathways (40). Given our dual goals to understand virus manipulation of cellular trafficking pathways and identify points of possible intervention, we chose to target a rationally selected set of cellular trafficking factors rather than conducting a genome-wide screen. As a proof of principle, all human Rab GTPases were screened previously for involvement in HSV1 morphogenesis, with four (Rab1, -6, -5, and -11) identified as being involved in the trafficking of virus glycoproteins and subsequent envelopment of the virus in endocytic membranes (7, 13). In this study, a range of 82 cellular trafficking factors covering different functions in the secretory and endocytic pathways were targeted, and 11 were identified whose depletion reduced infectious virus production by >20-fold, with an additional 14 reducing virus production by >10-fold, indicating a potential role for these proteins in HSV1 infection. This outcome compares favorably with the results of a recent study on the betaherpesvirus human cytomegalovirus (hCMV), where a screen of 156 host factors involved in membrane organization found that the depletion of 15 factors reduced virus yields between 5- and 12-fold (41). Moreover, a number of genome-wide RNA interference (RNAi) screens of different enveloped virus infections have all identified ∼300 targets out of ∼20,000 genes tested (42–45), indicating that our targeted screen was efficient in discovering important factors.

Of the five trafficking factors that were investigated in detail, two were found to inhibit the virus life cycle at early stages of infection prior to morphogenesis. First, the depletion of the COPG1 coatomer subunit γ1, which is one of seven subunits that form the stable coat complex COPI (21), was found to have the most profound effect on virus production in both library screens. COP1 coats form on vesicles and tubules and are responsible for the retrieval of proteins from the Golgi apparatus to the ER (46). COP1 also localizes to endosomes, but its role there is unclear (47). Not surprisingly, COP1 appears to be exploited by many viruses throughout their life cycles (48) and has recently been shown to be required for hCMV infection using an siRNA screen similar to the one described here (41). Nonetheless, although a potential role for COPG1 downstream in virus infection/trafficking of virus glycoproteins through the secretory pathway cannot be ruled out, the data presented here show that the predominant effect of COPG1 depletion is the inhibition of virus entry, potentially by reducing the level and presentation of the HSV1 receptor nectin1 at the plasma membrane. It should also be noted that COPG1 depletion affected the viability of cells, which in turn could reduce their ability to support early stages of virus infection (Fig. 1D). Second, the depletion of the v-SNARE protein VAMP4 had a profound effect on HSV1 genome replication, upstream of virus morphogenesis, and as a consequence, VAMP4-depleted cells supported a reduced level of late protein synthesis. Given that VAMP4 is enriched in the TGN and cycles from the cell surface to the TGN (25), this may suggest that rather than directly affecting glycoprotein trafficking, its depletion results in a global perturbation of cell integrity similar to that seen for COPG1 depletion, as evidenced by the reduced viability of VAMP4-depleted cells (Fig. 1D). This global effect may therefore specifically affect the process of genome trafficking or genome replication in the nucleus. In short, while both these factors have important roles in intracellular trafficking, neither can be assigned a direct role in HSV morphogenesis.

Three of the factors that were investigated were shown to perturb HSV1 morphogenesis specifically. AP4E1 is a late secretory pathway protein and is a component of the adaptor protein complex AP4, a poorly characterized complex involved in non-clathrin-coat vesicle formation and cargo selection on vesicles departing the TGN (22). AP4 is present at a relatively low abundance but is ubiquitously expressed and has been proposed to be involved in the trafficking of specialized cargoes, such as the transport of ATG9 to autophagosomes (49, 50). Although speculative at this stage, it is possible that HSV1 utilizes AP4 to transport one or more of its glycoproteins out of the TGN, which may explain the reduced localization of gD and gE at the cell surface in AP4E1-depleted infected cells (Fig. 4A), and further work will be required to determine if any glycoproteins contain functional AP4-binding motifs. The final two factors of the five tested, CHMP4C and STX10, were both found to localize to and be required for the integrity of recycling endosomes. This was unexpected for STX10, which has been shown previously to localize to the TGN (24, 32). Nonetheless, it is also required for recycling the transferrin receptor to the plasma membrane (32), a result that was confirmed here, placing its role within the recycling endocytic network and suggesting that its colocalization with the transferrin receptor in recycling endosomes may be functionally relevant. CHMP4C, on the other hand, is a component of the ESCRT-III fission machinery involved in membrane remodeling during various processes within the cell, including late endosomal sorting, plasma and nuclear membrane repair, and abscission during cytokinesis (36). However, it has not been previously located on recycling endosomes or shown to be required for their biogenesis. Among the other factors identified as reducing virus yield and not examined further (Table 1), the coat protein clathrin light chain A, while not being required for the formation of clathrin coats at the plasma membrane or the TGN, is required for coat formation in tubular endosomes involved in recycling to the plasma membrane (51). Additionally, dynamin, which was also identified as a hit in the second siRNA screen, is involved in the recycling of the transferrin receptor to the plasma membrane by the above-mentioned endosome-derived clathrin-coated vesicles (52). Collectively, these results identify multiple factors involved in the biogenesis of recycling endosomes that are important for HSV1 infection, adding weight to the importance of the recycling endocytic network in the morphogenesis of HSV1.

CHMP4 is the most abundant component of the ESCRT-III membrane-remodeling machinery and has a role in the envelopment of human immunodeficiency virus (HIV) (53), along with other viruses such as dengue virus (54). While there are three paralogues of CHMP4, the efficient depletion of CHMP4A or CHMP4B in isolation or in combination had no effect on HSV1 production here, indicating that the perturbation of HSV1 morphogenesis was highly specific to CHMP4C depletion (Fig. 5). This is in contrast to the situation with HIV budding, where CHMP4B but not -A or -C is required (53), and indeed, this differs from the results of a previous study on HSV1 that identified CHMP4B as being important for virus morphogenesis (34). However, in that study, the CHMP4 paralogues were not depleted, but instead, dominant negative CHMP4 proteins were overexpressed, indicating that all three dominant negative CHMP4 paralogues interfered with HSV1 production but with a greater effect of CHMP4B overexpression. While there is an apparent discrepancy with our results presented here, it should be noted that no evidence was provided in that study to show that the overexpression of each dominant negative paralogue was specific to the activity of its counterpart without interfering with the activity of the other paralogues. It is also at odds with another study that used the overexpression of dominant negative CHMP4, resulting in a greater effect of CHMP4C than of CHMP4B dominant negative expression on HSV1 production (55). A recent study has also proposed a role for ESCRT-III in the nuclear egress of HSV1 capsids (56). In that case, CHMP4B knockout caused a 5- to 10-fold drop in virus titers, whereas the depletion of all three paralogues together resulted in a 100-fold reduction in virus titers. However, CHMP4C depletion in isolation was not tested, and it is therefore not possible to judge the contribution made by the loss of CHMP4C in that study. Moreover, while those authors primarily reported a role for ESCRT-III at the nuclear envelope, a major effect of the absence of all three CHMP4 paralogues was the accumulation of naked capsids in the cytoplasm, similar to our results presented here. Nonetheless, although four different CHMP4C siRNAs resulted in a reduction of HSV1 production in our study here, it is still formally possible that our results were a consequence of off-target RNAi effects. Hence, in the future, it will be necessary to study HSV1 infection in cells knocked out rather than depleted for CHMP4C and to further ensure that complementation of these cells restores HSV1 yields to normal levels.

**FIG 9:**
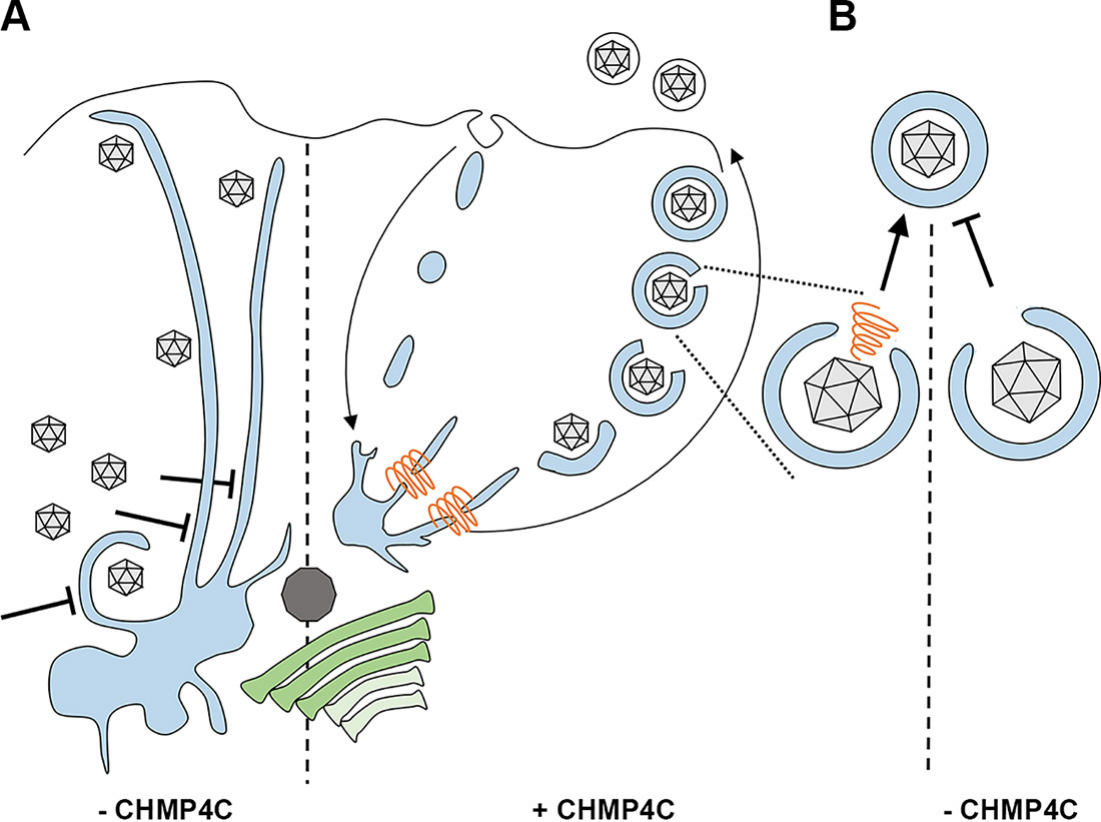
Possible models for the role of CHMP4C in HSV1 envelopment. (A) In the presence of CHMP4C (orange), recycling endocytic membranes (pale blue) are retrieved from the plasma membrane and utilized for virus envelopment by trafficking via the MTOC (dark gray) located close to the Golgi apparatus (green). This encloses the capsid in a double membrane, with the outer membrane being lost as it fuses with the plasma membrane during egress, to release a single enveloped capsid. In the absence of CHMP4C, the homeostasis of the ERC is altered by a failure of these endosomal membranes to undergo scission, similar to that seen previously (59), resulting in an accumulation of endocytic membranes at the MTOC and long tubules reaching to the cell periphery. (B) In the alternative scenario, CHMP4C is directly involved in closing the double membrane of the enveloping capsid, in a process analogous to the closure of autophagosomes. In the absence of CHMP4C, this closure fails or is slowed, resulting in reduced virion production.

Although ESCRT-III is generally considered to be involved in the scission of cytosol-containing membranes, such as HIV budding or cytokinesis, there is also increasing evidence for a role for this machinery in the involution of membranes that exclude cytosol, as would be the case in the scission of endocytic membranes (57). For example, the formation of peroxisomes at the ER in Saccharomyces cerevisiae has been shown to involve snf7, the yeast counterpart of CHMP4 (58). Of note, for the results presented here, ESCRT-III has also been shown to promote the fission of endocytic tubules from endosomal membranes (59). The data presented here suggest that in at least some situations, and specifically in HSV1 infection, CHMP4C is required for the biogenesis of recycling endosomes. While less pronounced in uninfected cells, recycling endosomes were aberrantly localized to the MTOC in HSV1-infected cells, with thin tubules emanating toward the periphery of the cell, indicating a perturbation to the normal trafficking of this compartment. Given that this network is proposed to be important in HSV1 envelopment (7), the profound effect of CHMP4C depletion together with HSV1 infection on the appearance of these membranes may indicate that HSV1 either specifically activates and utilizes a population that requires CHMP4C for correct scission or redirects CHMP4C for this process. Extensive tubulation of recycling endosomes has been detected in other related scenarios, such as BFA-mediated inhibition of Arf GTPases, required for coat formation and budding from membranes (38); knockdown of endosome-localized Arf GTPases themselves (60); and knockdown of the BIG2 guanidine exchange factor (GEF) for such Arfs (61). Although it is not yet known if this tubulation represents abrogated fission of these membranes on the way to the ERC from sorting endosomes or on the way out of the ERC on the way to the plasma membrane (62), it is clear that the tubulation/accumulation phenotype represents a defect in the biogenesis of recycling endosomes. This suggests that HSV1 envelopment could be inhibited in the absence of CHMP4C due to failed scission of endocytic membranes, resulting in the depletion of the pool of available wrapping membranes (Fig. 9A). Alternatively, the capsid wrapping profiles that were detected in CHMP4C-depleted infected cells, whereby the capsid was observed in association with a cuplike double membrane that had not yet sealed to form a doubly enveloped virion (Fig. 7H), could reflect a requirement for CHMP4C in the final closure of the neck of this structure (Fig. 9B), a process that would share topology with canonical ESCRT-III-dependent processes (36) such as the sealing of the phagophore in autophagy (63). Of note, CHMP2A, which was shown recently to regulate phagophore closure (64), was also picked up here in our second library screen as a possible factor involved in HSV1 infection (Table 1).

It is becoming increasingly accepted that host cell processes required for virus infection could be appropriate targets for antiviral intervention (65, 66), and virus morphogenesis and envelopment may offer an opportunity for such intervention. Moreover, the study of virus morphogenesis provides the opportunity to identify functions of as-yet-uncharacterized cellular trafficking proteins. Our discovery of a new role for CHMP4C in the biogenesis of HSV1-wrapping membranes paves the way for future work in both areas.

## MATERIALS AND METHODS

### Cells and viruses.

Vero and HeLa cells were cultured in Dulbecco’s modified Eagle’s medium (DMEM) supplemented with 50 U/ml penicillin/streptomycin and 10% fetal bovine serum (FBS). All viruses were routinely propagated in Vero cells in DMEM supplemented with 2% newborn calf serum (NCS) and 50 U/ml penicillin/streptomycin. All plaque assays were carried out in Vero cells in DMEM supplemented with 2% NCS, 50 U/ml penicillin/streptomycin, and 1% human serum (BioIVT). HSV1 strain Sc16 was used routinely. Sc16 110lacZ (29) was previously described and was provided by Stacey Efstathiou (University of Cambridge).

### siRNAs and transfections.

Silencer Select siRNA duplexes (Ambion, Thermo Fisher Scientific) were forward or reverse transfected with Lipofectamine 2000 (Invitrogen) in HeLa cells to a final concentration of 20 nM according to the manufacturer’s instructions. Silencer Select negative-control siRNA 1 was used as a negative control (Ambion, Thermo Fisher Scientific). The identification numbers of specific siRNAs used were s40954 (CHMP4C), 38462 (CHMP4C-A), 38373 (CHMP4C-B), 127955 (CHMP4C-C), s26441 (CHMP4A), s43363 (CHMP4B), s22431 (COPG1), s23818 (AP4E1), s16535 (STX10), s16526 (VAMP4), and s11605 (nectin1).

### Plasmids.

CHMP4C and STX10 open reading frames were amplified by PCR from HeLa cDNA using primers shown in Table S5 in the supplemental material and inserted into the pCR-BluntII-TOPO cloning plasmid. CHMP4C and STX10 open reading frames were transferred as BamHI-EcoRI and BamHI-BamHI fragments, respectively, into an in-house expression vector driven by the cytomegalovirus IE (CMV-IE) promoter (67), which places the V5 tag at the N termini of both proteins. Nectin1A was first amplified from HeLa cDNA using primer set 1 shown in Table S5 and inserted into the pCR-BluntII-TOPO cloning plasmid. The nectin1A open reading frame was subsequently amplified by PCR using primer set 2 and inserted into pEGFPN1 as a BamHI-NotI fragment, incorporating the V5 epitope tag at the C terminus of nectin1A.

10.1128/mBio.02183-20.8 TABLE S5 PCR primers for amplification and cloning from HeLa cell cDNA. Bold, underlined sequences are restriction sites used for cloning. Lowercase type indicates the V5 epitope sequence. Download Table S5, DOCX file, 0.02 MB. Copyright © 2021 Russell et al. 2021 Russell et al. https://creativecommons.org/licenses/by/4.0/ This content is distributed under the terms of the Creative Commons Attribution 4.0 International license.

### Antibodies and reagents.

The following primary antibodies were used for Western blotting and immunofluorescence and were kindly provided: mouse anti-gD (LP14) and mouse anti-gB (R69), from Tony Minson (University of Cambridge); mouse anti-VP16 (LP1), from Colin Crump (University of Cambridge); mouse anti-gE, from David Johnson (Oregon Health and Science University); and mouse anti-nectin1 (CK6), from Claude Krummenacher (Rowan University, NJ). Our rabbit VP22-specific antibody (AGV031) was described previously (68). Commercially available antibodies used in this study included mouse anti-V5, mouse anti-gC, mouse anti-β-actin, mouse anti-CD63, rabbit anti-giantin, mouse anti-CHMP4A, and rabbit anti-CHMP4B (all from Abcam); mouse anti-CD71 (Santa Cruz); and mouse anti-γ-tubulin and mouse anti-α-tubulin (Sigma). Goat anti-mouse IRDye 680RD and goat anti-rabbit IRDye 800CW (Li-Cor Biosciences) secondary antibodies were used as appropriate for Western blotting, while Alexa Fluor-conjugated secondary antibodies (Invitrogen) were used for immunofluorescence. Endocytic structures were labeled by incubating cells with Texas Red-conjugated transferrin (Invitrogen) or Alexa Fluor 488-conjugated transferrin (Molecular Probes) at a concentration of 1 μg/ml for 30 min. Brefeldin A (Sigma) was used at a concentration of 1 μg/ml.

### Viability assay.

Cell viability was assessed using a CellTiter-Glo luminescent viability assay (Promega) and read on a CLARIOstar microplate reader (BMG Labtech).

### SDS-PAGE and Western blotting.

Samples were separated by SDS-PAGE (between 10 and 14% polyacrylamide as appropriate) and transferred to nitrocellulose membranes before blots were imaged using an Odyssey CLx imaging system (Li-Cor Biosciences).

### N-linked glycosylation analysis.

Cell lysates were lysed on ice in glycosylation lysis buffer (50 mM Tris base [pH 7.5], 200 mM sodium chloride, 2 mM magnesium chloride, and 1% [vol/vol] NP-40) with a protease inhibitor cocktail (Thermo Fisher). Glycoprotein denaturation of the cell lysate was performed with 1 × glycodenaturing buffer (New England BioLabs) at 95°C for 10 min. The denatured cell lysate was then deglycosylated using 500 U of PNGase F (New England BioLabs) in 1× glycobuffer 2 (New England BioLabs) and 10% NP-40 (New England BioLabs) at 37°C for 60 min.

### Transmission electron microscopy.

To prepare samples for electron microscopy, cells were grown to approximately 50% confluence before transfection with siRNA duplexes to a final concentration of 20 nM. After 48 h, cells were then infected with HSV1 Sc16 at a multiplicity of infection (MOI) of 5 for 12 h. Endocytic events were labeled by incubating cells with medium containing transferrin-HRP (Jackson ImmunoResearch) at a concentration of 1 μg/ml for 30 min prior to fixation in 0.5% glutaraldehyde in 200 mM sodium cacodylate buffer for 30 min before being washed in sodium cacodylate buffer. After fixation, samples were washed and stained with a metal-enhanced 3,3′-diaminobenzidine (DAB) substrate kit (Thermo Fisher Scientific). The samples for electron microscopy were fixed and processed as described previously (30).

### RNA isolation, reverse transcription, and qPCR.

RNA was isolated from cells using an RNeasy minikit (Qiagen), and 400 ng to 1 μg of RNA was then DNase I treated according to the manufacturer’s protocol (Thermo Fisher Scientific). Superscript III (Invitrogen) was used with a random primer mix to make cDNA according to the manufacturer’s instructions in a Veriti 96-well thermal cycler (Applied Biosystems). All quantitative PCRs (qPCRs) were assembled using the Mesa Blue qPCR kit for SYBR assay (Eurogentec) according to the manufacturer’s instructions, with the primer sets listed in Table S4. The qPCRs were carried out on a LightCycler96 system (Roche).

10.1128/mBio.02183-20.7 TABLE S4 RT-qPCR primers used for measuring siRNA knockdown efficiency. Download Table S4, DOCX file, 0.02 MB. Copyright © 2021 Russell et al. 2021 Russell et al. https://creativecommons.org/licenses/by/4.0/ This content is distributed under the terms of the Creative Commons Attribution 4.0 International license.

### Quantification of viral DNA.

HeLa cells were reverse transfected with 20 nM siRNA duplexes and incubated for 48 h before infection with HSV1 Sc16 at an MOI of 2, including a control of untransfected cells infected in the presence of 100 ng/ml cytosine arabinoside (AraC). After 1 h, cells were subjected to a gentle acid wash to inactivate any virus that had not entered the cells. After a total of 2 or 24 h of infection, DNA was harvested using the DNeasy blood and tissue kit (Qiagen). The qPCR assays were carried out in a LightCycler96 system (Roche) using the Mesa Blue qPCR kit for SYBR assay (Eurogentec) according to the manufacturer’s instructions with primers for 18S (Table S4) and the HSV1 *UL48* gene.

### Immunofluorescence.

Cells for immunofluorescence were grown on coverslips and fixed with 4% paraformaldehyde in phosphate-buffered saline (PBS) for 20 min at room temperature, followed by permeabilization with 0.5% Triton X-100 for 10 min. Fixed cells were blocked by incubation in PBS with 10% NCS for 20 min, before the addition of primary antibody in PBS with 10% NCS, and a further 30-min incubation. After extensive washing with PBS, the appropriate Alexa Fluor-conjugated secondary antibody was added in PBS with 10% NCS and incubated for a further 30 min. The coverslips were washed extensively in PBS and mounted in Vectashield (Vector Labs) containing 4′,6-diamidino-2-phenylindole (DAPI). Images were acquired using a Nikon A1 confocal microscope and processed using ImageJ software (69).
