# Supplementary material for: Novel Role for ESCRT-III Component CHMP4C in the Integrity of the Endocytic Network Utilized for Herpes Simplex Virus Envelopment
Source: mBio. 2021 May 11;12(3):e02183-20. doi: 10.1128/mBio.02183-20 (PMC8262985; doi:10.1128/mBio.02183-20)
Supplement: TABLE S1 [file mbio.02183-20-st001.docx]

**Table S1.** Genes for human trafficking factors targeted by siRNA library

| **Factor** | **Official HUGO Name** | **Symbol** | **NCBI Entrez Gene ID** |
| --- | --- | --- | --- |
| **COAT PROTEINS** | clathrin light chain A | CLTA | 1211 |
|  | clathrin light chain B | CLTB | 1212 |
|  | clathrin heavy chain | CLTC | 1213 |
|  | secretion associated Ras related GTPase 1A | SAR1A | 56681 |
|  | secretion associated, Ras related GTPase 1B | SAR1B | 51128 |
|  | Sec23 homolog A, coat complex II component | SEC23A | 10484 |
|  | Sec23 homolog B, coat complex II component | SEC23B | 10483 |
|  | SEC24 homolog A, COPII coat complex component | SEC24A | 10802 |
|  | SEC24 homolog B, COPII coat complex component | SEC24B | 10427 |
|  | SEC24 homolog C, COPII coat complex component | SEC24C | 9632 |
|  | SEC24 homolog D, COPII coat complex component | SEC24D | 9871 |
|  | SEC13 homolog, COPII coat complex component | SEC13 | 6396 |
|  | coatomer protein complex subunit gamma 1 | COPG1 | 22820 |
|  | coatomer protein complex subunit gamma 2 | COPG2 | 26958 |
| **ADAPTOR PROTEINS** | adaptor related protein complex 1 beta 1 subunit | AP1B1 | 162 |
|  | adaptor related protein complex 1 mu 1 subunit | AP1M1 | 8907 |
|  | adaptor related protein complex 1 mu 2 subunit | AP1M2 | 10053 |
|  | adaptor related protein complex 1 sigma 1 subunit | AP1S1 | 1174 |
|  | adaptor related protein complex 1 sigma 2 subunit | AP1S2 | 8905 |
|  | adaptor related protein complex 1 sigma 3 subunit | AP1S3 | 130340 |
|  | adaptor related protein complex 1 gamma 1 subunit | AP1G1 | 164 |
|  | adaptor related protein complex 1 gamma 2 subunit | AP1G2 | 8906 |
|  | adaptor related protein complex 2 alpha 1 subunit | AP2A1 | 160 |
|  | adaptor related protein complex 2 alpha 2 subunit | AP2A2 | 161 |
|  | adaptor related protein complex 2 beta 1 subunit | AP2B1 | 163 |
|  | adaptor related protein complex 2 mu 1 subunit | AP2M1 | 1173 |
|  | adaptor related protein complex 2 sigma 1 subunit | AP2S1 | 1175 |
|  | adaptor related protein complex 3 beta 1 subunit | AP3B1 | 8546 |
|  | adaptor related protein complex 3 beta 2 subunit | AP3B2 | 8120 |
|  | adaptor related protein complex 3 delta 1 subunit | AP3D1 | 8943 |
|  | adaptor related protein complex 3 mu 1 subunit | AP3M1 | 26985 |
|  | adaptor related protein complex 3 mu 2 subunit | AP3M2 | 10947 |
|  | adaptor related protein complex 3 sigma 1 subunit | AP3S1 | 1176 |
|  | adaptor related protein complex 3 sigma 2 subunit | AP3S2 | 10239 |
|  | adaptor related protein complex 4 beta 1 subunit | AP4B1 | 10717 |
|  | adaptor related protein complex 4 mu 1 subunit | AP4M1 | 9179 |
|  | adaptor related protein complex 4 epsilon 1 subunit | AP4E1 | 23431 |
|  | adaptor related protein complex 4 sigma 1 subunit | AP4S1 | 11154 |
| **FISSION FACTORS** | dynamin 2 | DNM2 | 1785 |
|  | dynamin 3 | DNM3 | 26052 |
|  | charged multivesicular body protein 6 | CHMP6 | 79643 |
|  | charged multivesicular body protein 4A | CHMP4A | 29082 |
|  | charged multivesicular body protein 4B | CHMP4B | 128866 |
|  | charged multivesicular body protein 4C | CHMP4C | 92421 |
|  | charged multivesicular body protein 3 | CHMP3 | 51652 |
|  | charged multivesicular body protein 2A | CHMP2A | 27243 |
|  | charged multivesicular body protein 2B | CHMP2B | 25978 |
| **FUSION FACTORS** | syntaxin 2 | STX2 | 2054 |
|  | syntaxin 3 | STX3 | 6809 |
|  | syntaxin 4 | STX4 | 6810 |
|  | syntaxin 5 | STX5 | 6811 |
|  | syntaxin 6 | STX6 | 10228 |
|  | syntaxin 7 | STX7 | 8417 |
|  | Syntaxin 8 | STX8 | 9482 |
|  | syntaxin 10 | STX10 | 8677 |
|  | syntaxin 11 | STX11 | 8676 |
|  | syntaxin 12 | STX12 | 23673 |
|  | syntaxin 16 | STX16 | 8675 |
|  | synaptosome associated protein 23kDa | SNAP23 | 8773 |
|  | synaptosome associated protein 29kDa | SNAP29 | 9342 |
|  | vesicle associated membrane protein 3 | VAMP3 | 9341 |
|  | vesicle associated membrane protein 4 | VAMP4 | 8674 |
|  | vesicle associated membrane protein 5 | VAMP5 | 10791 |
|  | vesicle associated membrane protein 7 | VAMP7 | 6845 |
|  | vesicle associated membrane protein 8 | VAMP8 | 8673 |
| **RAB1 EFFECTORS** | USO1 vesicle transport factor | USO1 | 8615 |
|  | Golgin A2 | GOLGA2 | 2801 |
|  | Golgin B1 | GOLGB1 | 2804 |
|  | Golgin A5 | GOLGA5 | 9950 |
|  | RUN and SH3 domain containing 2 | RUSC2 | 9853 |
|  | microtubule associated monooxygenase, calponin and LIM domain containing 1 | MICAL1 | 64780 |
|  | microtubule associated monooxygenase, calponin and LIM domain containing 2 | MICAL2 | 9645 |
|  | microtubule associated monooxygenase, calponin and LIM domain containing 3 | MICAL3 | 57553 |
| **RAB5 EFFECTORS** | rabaptin, RAB GTPase binding effector protein 1 | RABEP1 | 9135 |
|  | early endosome antigen 1 | EEA1 | 8411 |
|  | rabenosyn, RAB effector | RBSN | 64145 |
|  | phosphatidylinositol 3-kinase catalytic subunit type 3 | PIK3C3 | 5289 |
|  | phosphoinositide-3-kinase regulatory subunit 1 | PIK3R1 | 5295 |
|  | phosphatidylinositol-4-phosphate 3-kinase catalytic subunit type 2 beta | PIK3C2B | 5287 |
|  | Rabip4' |  |  |
|  | ankyrin repeat and FYVE domain containing 1 | ANKFY1 | 51479 |
|  | coagulation factor VIII-associated 1 | F8A1 | 8263 |
