# Supplementary material for: Novel Role for ESCRT-III Component CHMP4C in the Integrity of the Endocytic Network Utilized for Herpes Simplex Virus Envelopment
Source: mBio. 2021 May 11;12(3):e02183-20. doi: 10.1128/mBio.02183-20 (PMC8262985; doi:10.1128/mBio.02183-20)
Supplement: FIG S1 [file mbio.02183-20-sf001.docx]

**
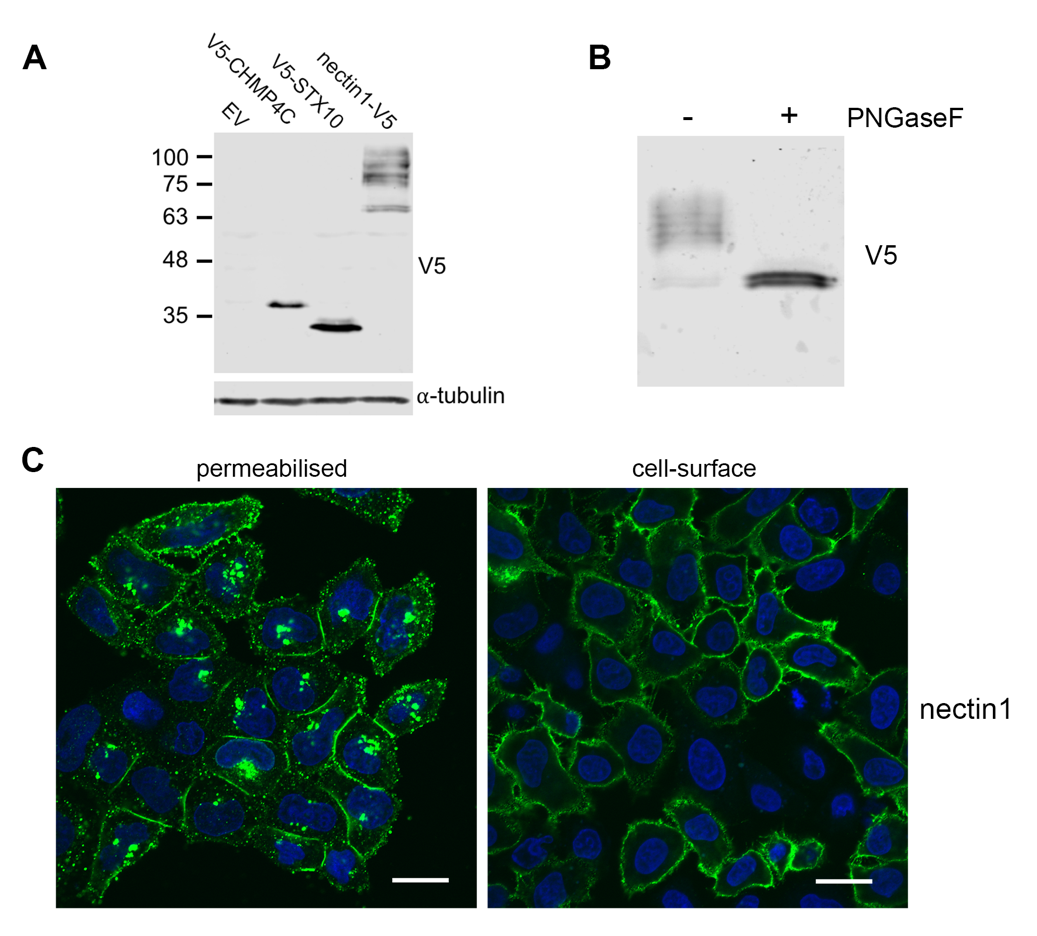
**

**Figure S1.** Transient expression of V5-tagged constructs used in this study. (**A**) HeLa cells were transfected with an empty vector (EV), or plasmids expressing V5-CHMP4C, V5-STX10 or nectin1-V5, and analysed 16 h later by SDS-PAGE and Western blotting with antibody to the V5 epitope and α-tubulin. (**B**) As for (**A**), but nectin1-V5 transfected cells were harvested and deglycosylated with PNGaseF prior to analysing by SDS-PAGE and Western blotting. (**C**) HeLa cells grown on coverslips were transfected with nectin1-V5-expressing plasmid. Sixteen hours later, cells were either cell-surface stained with antibody to the extracellular domain of nectin1 prior to fixation, or fixed and permeabilised followed by staining with the same antibody (green). Nuclei were stained with DAPI (blue). Scale bar = 20 μm.
