# Supplementary material for: Novel Role for ESCRT-III Component CHMP4C in the Integrity of the Endocytic Network Utilized for Herpes Simplex Virus Envelopment
Source: mBio. 2021 May 11;12(3):e02183-20. doi: 10.1128/mBio.02183-20 (PMC8262985; doi:10.1128/mBio.02183-20)
Supplement: FIG S2 [file mbio.02183-20-sf002.docx]

**
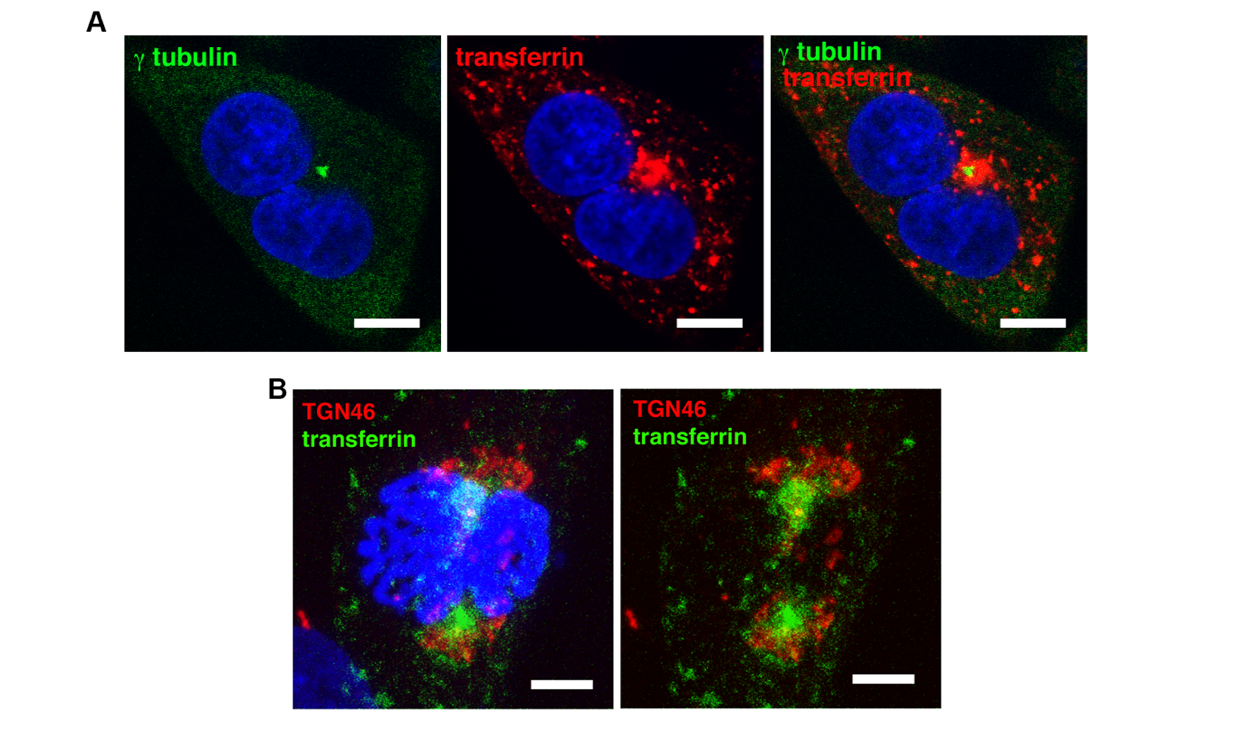
**

**Figure S2.** Uptake of transferrin into HeLa cells. (**A**) HeLa cells were incubated with texas red conjugated transferrin for 30 min, before fixing and staining for γ tubulin (green) to label the MTOC. (**B**) HeLa cells were incubated with FITC-transferrin (green) for 30 mins, before fixing and staining for TGN46 (red). Scale bar = 5 μM.­­
