# Supplementary material for: Novel Role for ESCRT-III Component CHMP4C in the Integrity of the Endocytic Network Utilized for Herpes Simplex Virus Envelopment
Source: mBio. 2021 May 11;12(3):e02183-20. doi: 10.1128/mBio.02183-20 (PMC8262985; doi:10.1128/mBio.02183-20)
Supplement: FIG S3 [file mbio.02183-20-sf003.docx]

**
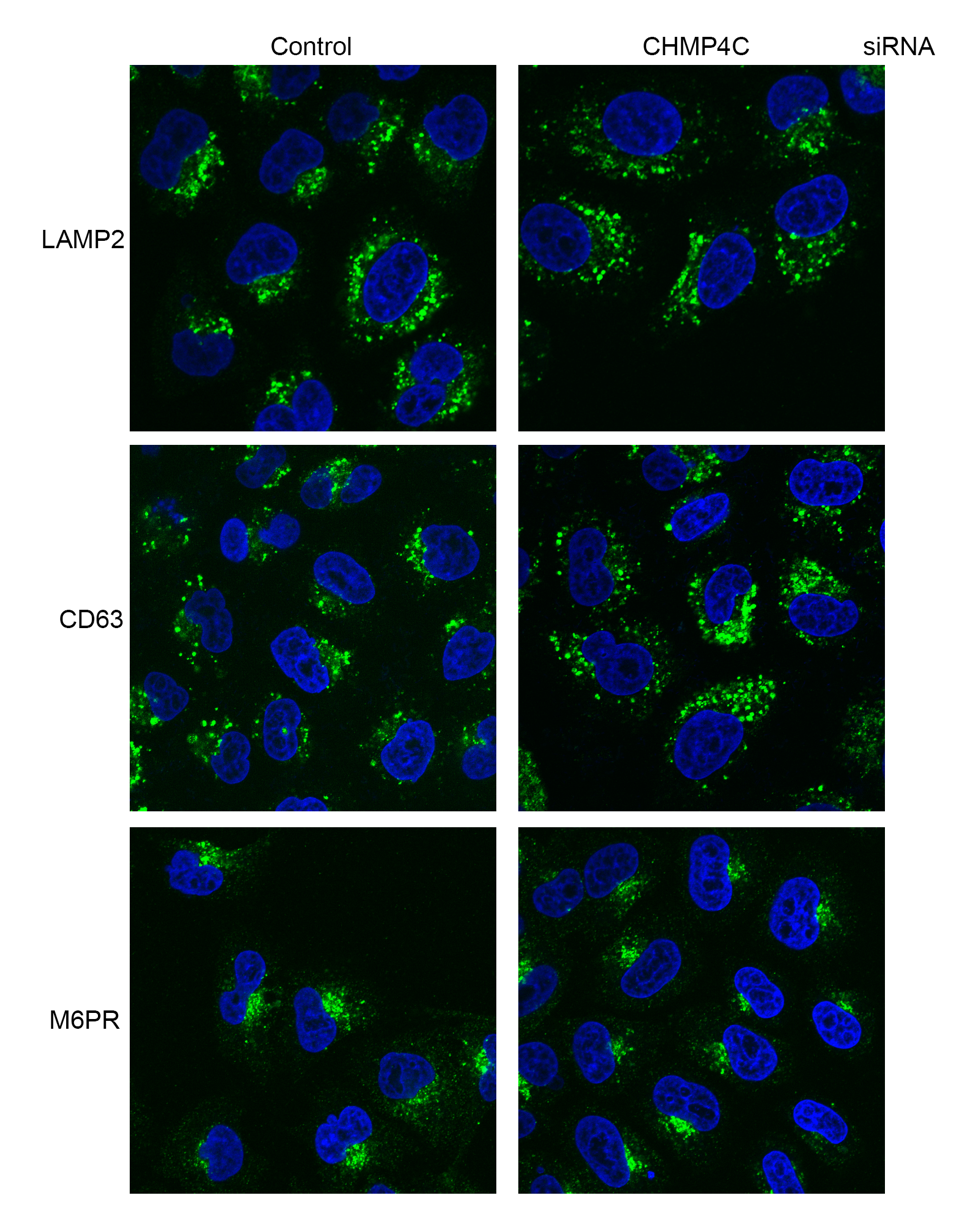
**

**Figure S3.** Depletion of CHMP4C has no effect on the late secretory pathway**.** HeLa cells were transfected with control or CHMP4C siRNAs and fixed and stained two days later for the lysosomal marker LAMP2, the late endosomal marker CD63 or the mannose 6 phosphate receptor M6PR.
