# Supplementary material for: Novel Role for ESCRT-III Component CHMP4C in the Integrity of the Endocytic Network Utilized for Herpes Simplex Virus Envelopment
Source: mBio. 2021 May 11;12(3):e02183-20. doi: 10.1128/mBio.02183-20 (PMC8262985; doi:10.1128/mBio.02183-20)
Supplement: TABLE S5 [file mbio.02183-20-st005.docx]

**Table S5.** PCR primers for amplification and cloning from HeLa cell cDNA. Bold, underlined sequences are restriction sites used for cloning. Lower case is V5 epitope sequence.

| **Target** | **Primer Sequence** | |
| --- | --- | --- |
|  | **Forward** | **Reverse** |
| CHMP4C | **GGATCC**ATGAGCAAGTTGGGCAAG | TTAGGTAGCCCAAGCTGCC |
| STX10 | TCT**GGATCC**ATGTCTCTCGAAGACCCCTT | GTT**GGATCC**TCAGAGAGAGAATAGTAAGATGAGAACG |
| Nectin1A  (set 1) | ATGGCTCGGATGGGGC | GTTGGATCCCTACACGTACCACTCCTTCTTGGA |
| Nectin1A  (set 2) | TCT**GGATCC**ACATGGCTCGGA | GC**GCGGCCGC**CTAggtggagtccaatcccagcaaagggtttgggatcggctttccCACGTACCACTCCTTCTTGG |
