# Supplementary material for: Novel Role for ESCRT-III Component CHMP4C in the Integrity of the Endocytic Network Utilized for Herpes Simplex Virus Envelopment
Source: mBio. 2021 May 11;12(3):e02183-20. doi: 10.1128/mBio.02183-20 (PMC8262985; doi:10.1128/mBio.02183-20)
Supplement: TABLE S4 [file mbio.02183-20-st004.docx]

**Table S4.** RT-qPCR primers used for measuring siRNA knockdown efficiency.

| **Target** | **Primer Sequence** | |
| --- | --- | --- |
|  | **Forward** | **Reverse** |
| AP4E1 | GGCTCTTCAACACCAGATGAC | CCAGCTCTGCTATTTTGCCG |
| COPG1 | GCAACACGCCGTCCTTATG | CACTGTGAACTCGCAGTCCT |
| CHMP4A | GAGCTCGCGATGAGTGGTC | TCTTGGCTGTTTGTAGCTCCT |
| CHMP4B | CAAGAAGCACGGCACCAAAA | GCCAGCTGCTTCTCATACCT |
| CHMP4C | TTCTCAACGGGTTGGCTTTG | GTGGACGACATGCCTGGTTT |
| STX10 | TGGACTGGACGACCAATGAG | GATGTAGCGAGATGTGGCCG |
| VAMP4 | TGAGAGAGGGGAGAGACTAGATG | GGATAGCAGCAACCAAAGCC |
| 18S | CCAGTAAGTGCGGGTCATAAGC | GCCTCACTAAACCATCCAATCGG |
| VP16 | TAACCGTCTCCTCGACGACT | CTGGGCAGCGTTGATAGGAA |
